# *Senecio polyanthemoides* Sch. Bip. (Asteraceae) Essential Oils: Chemical Composition, Interpopulation Variability Study and In Vitro Biological Activities of Eight Wild Populations

**DOI:** 10.3390/molecules31122006

**Published:** 2026-06-08

**Authors:** Kehinde O. Amisu, Oladipupo A. Lawal, Olufemi A. Giwa-ajeniya, Victoria A. Dada, Kanyinsola O. Akinkunmi, Omobolanle E. Oladapo, Oyinlade C. Ogundare, Isyaku Bello, Emmanuel E. Aduak, Foluso O. Osunsanmi, Rebamang A. Mosa, Mona M. E. Eleiwa, Andy R. Opoku, Adebola O. Oyedeji

**Affiliations:** 1 Department of Microbiology, Lagos State University, PMB 0001 LASU Post Office, Ojo, Lagos State, Nigeria; kehinde.amisu@lasu.edu.ng (K.O.A.); oladapoomobola@gmail.com (O.E.O.); 2 Department of Chemistry, University of Zululand, Private Bag X1001, KwaDlangezwa 3886, South Africa; oyedejio@unizulu.ac.za; 3 Department of Chemistry, Lagos State University, PMB 0001 LASU Post Office, Ojo, Lagos State, Nigeria; femi.giwa-ajeniya@lasu.edu.ng (O.A.G.-a.); victoriadada0409@gmail.com (V.A.D.); akinkunmikanyinsola@gmail.com (K.O.A.); ishaqmuhdbello@gmail.com (I.B.); emmanueladuak01@gmail.com (E.E.A.); 4 Department of Microbiology, Lagos State College of Nursing, Alimosho General Hospital Complex, LASU/Isheri Road, Igando, Lagos State, Nigeria; 5 Department of Chemical Sciences, College of Basic Science, Lagos State University of Science and Technology, PMB 1004, Ikorodu, Lagos State, Nigeria; ogundare.oc@lasustech.edu.ng; 6 School of General Studies, Jigawa State College of Education, POB 1002, Gumel 732102, Jigawa State, Nigeria; 7 Department of Biochemistry & Microbiology, University of Zululand, Private Bag X1001, KwaDlangezwa 3886, South Africa; alafin21@yahoo.com (F.O.O.); rebamang.mosa@up.ac.za (R.A.M.); opokua@unizulu.ac.za (A.R.O.); 8 Department of Biochemistry, Genetics and Microbiology, University of Pretoria, Private Bag X20, Hatfield 0028, Pretoria 0002, South Africa; 9 Department of Biology, Ibn Sina National College for Medical Studies, P.O. Box 31906, Al Mahjar, Jeddah 21418, Saudi Arabia; eleiwa_el_m@yahoo.com

**Keywords:** *Senecio polyanthemoides*, Asteraceae, essential oil, chemotypes, variability, biological activity

## Abstract

The in vitro antibacterial, antioxidant, cytotoxic and larvicidal activities of essential oils isolated by hydrodistillation from the aerial parts (above the ground organs consisting of the leaves, stems and flowers) of *Senecio polyanthemoides* growing in three district municipalities of KwaZulu-Natal Province, South Africa were investigated. The water-distilled oils were analyzed by GC and GC/MS. Sixty-three constituents were characterized in all the samples analyzed representing 86.0–99.2% of the total oil compositions. The major components of the oils were monoterpenoid compounds with β-pinene, myrcene, α-phellandrene, *cis*-β-ocimene, *trans*-β-ocimene, β-caryophyllene, α-humulene and germacrene D found in all samples. Numerical cluster and principal component analyses of the major constituents revealed four well-defined chemotypes and a high variability within the oil samples. The essential oils show significant multi-methods activities on antioxidant, cytotoxic, and larvicidal, alongside effective antibacterial action against some key pathogens (*Staphylococcus aureus*, *Escherichia coli*, *Bacillus pumilus* and *Enterococcus faecalis*), with inhibition zone ranging from (8.3 ± 0.6–27.3 ± 0.9) mm and MIC values of ≤5.0 mg/mL. Documenting the inaugural, in-depth analysis of *S. polyanthemoides* growing in South Africa, this study provides novel findings on its antioxidant, larvicidal, brine shrimp lethality, and antibacterial activities.

## 1. Introduction

The genus *Senecio* comprises over 1500 species distributed worldwide and has been extensively investigated for its diverse medicinal properties, including antimicrobial, anti-inflammatory, and antioxidant activities [1,2]. Historically, several *Senecio* species have been widely used in traditional medicine for the treatment of a variety of ailments, including respiratory infections, wounds, inflammatory disorders, and gastrointestinal diseases [3,4,5]. In southern Africa, some *Senecio* species are widely used ethnomedicinally by indigenous communities [4,5], and these traditional applications are supported by phytochemical studies that have identified a wide range of secondary metabolites, including pyrrolizidine alkaloids, flavonoids, sesquiterpenes, diterpenes, and triterpenoids, which contribute significantly to their pharmacological potential [3,5].

*Senecio polyanthemoides* Sch. Bip. (Asteraceae) is a species widely distributed in South Africa, where it is typically associated with grassland and forest-margin ecosystems [4]. It occurs extensively in the KwaZulu-Natal Province and is commonly found in disturbed environments and open vegetation types [3]. Despite its broad geographic distribution and notable ecological adaptability [4,6], *S. polyanthemoides* remains comparatively underexplored relative to other species within the genus. Existing studies on *S. polyanthemoides* have largely concentrated on its essential oil profile [7], which is dominated by monoterpenes and sesquiterpenes. Reported major constituents include limonene, *p*-cymene, α-pinene, β-pinene, 1,8-cineole, and caryophyllene oxide. Many of these compounds are recognized for their antimicrobial, antioxidant, and anti-inflammatory activities, indicating that the species may hold considerable biological and pharmacological relevance [8,9].

Although the genus *Senecio* has attracted increasing scientific attention in recent years [7,8,9,10,11], information on *S. polyanthemoides* across different geographical regions remains limited. This gap in knowledge highlights the need for further investigation to better understand the species’ phytochemical diversity, as well as its potential pharmacological properties and applications.

In continuation of our ongoing research program focused on the characterization of the chemical composition and biological activities of essential oils derived from South African flora [7,10,11], the present study was undertaken to address this deficiency. Specifically, this work provides a comprehensive evaluation of the essential oils obtained from the aerial parts of *S. polyanthemoides*, with emphasis on their chemical composition, interpopulation variability, and bioactivities, including (antioxidant, larvicidal, brine shrimp lethality, and antibacterial).

The novelty of this study lies in its integrative approach, which combines comparative analysis across multiple populations, assessment of chemical variability, and correlation of phytochemical profiles with biological activities, thereby offering new insights into intraspecific variation within the species. Overall, the findings aim to expand the current understanding of *S. polyanthemoides* and to support its potential as a promising source of bioactive compounds for pharmaceutical and therapeutic applications.

## 2. Results and Discussion

### 2.1. Essential Oil Compositions

The essential oils obtained from the aerial parts of *S. polyanthemoides* collected from eight different locations offered a golden brown to pale and golden yellow oils with the yields and refractive indexes ranging from 0.10 to 0.13% (based on fresh weight) and (1.4772–1.5292), respectively (Table 1).

**Figure 1:**
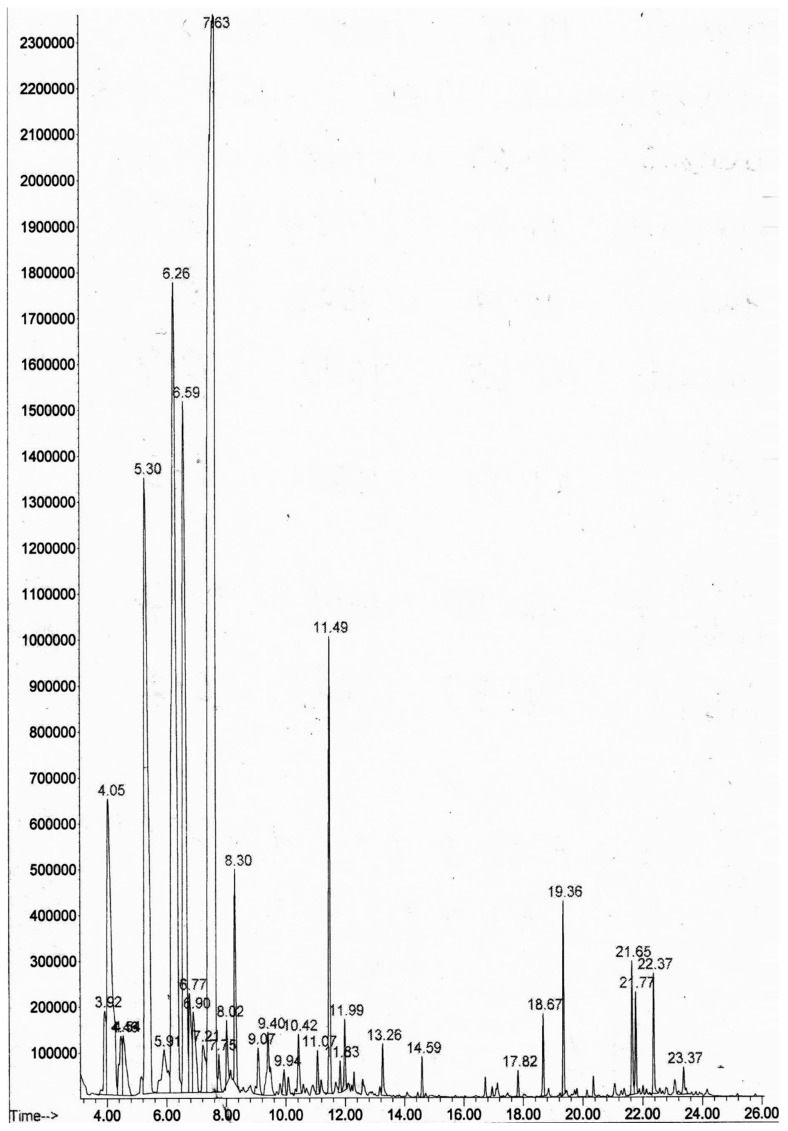
GC/MS Chromatogram of *S. polyanthemoides* (S4).

**Figure 2:**
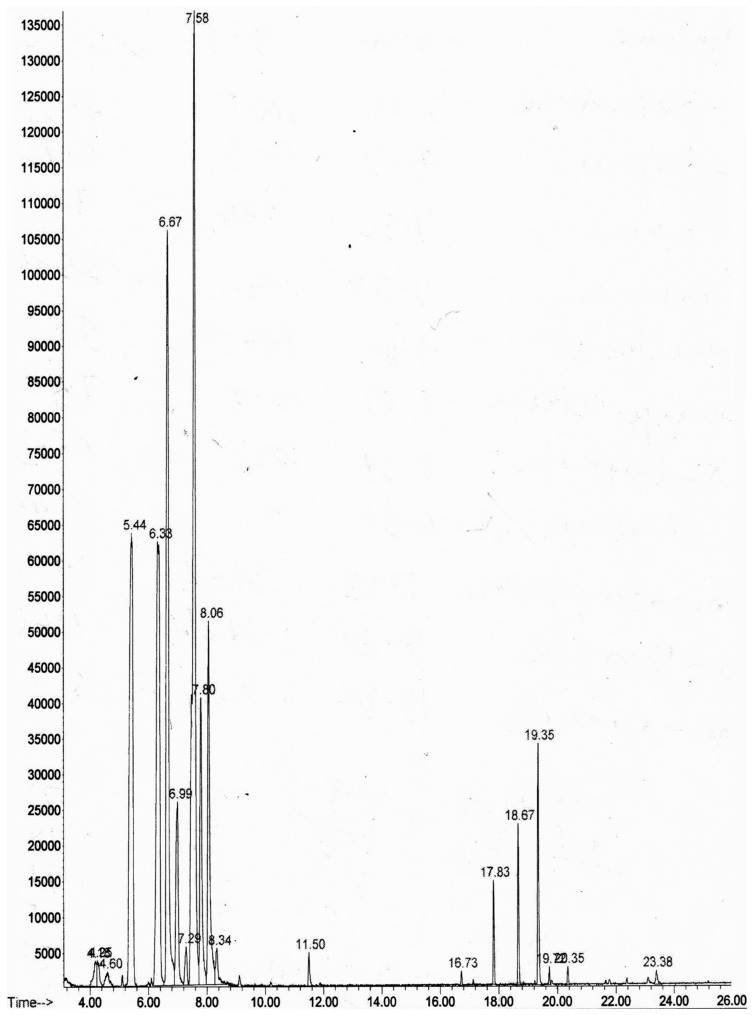
GC/MS Chromatogram of *S. polyanthemoides* (S6).

The qualitative and quantitative profiles of the essential oils extracted from *S. polyanthemoides* reveal a complex mixture of volatile constituents. Representative GC-MS chromatograms for samples 4 and 6 (Figure 1 and Figure 2, respectively) illustrate chemical profiles heavily dominated by monoterpene hydrocarbons. Analysis of the compositional data in Table 2 confirms that these monoterpenes constitute the bulk of the oil, whereas sesquiterpene hydrocarbons, oxygenated monoterpenes, and oxygenated sesquiterpenes are present only as minor components. Among the major compounds consistently detected were α-pinene, limonene, *p*-cymene, β-caryophyllene, spathulenol, and caryophyllene oxide, though their relative abundances varied considerably across samples.

This pronounced variation in the relative proportions of the dominant constituents indicates a high degree of intraspecific chemical polymorphism. Similar variability has been reported previously for *S. polyanthemoides*, where limonene (3.1–43.0%), *p*-cymene (4.9–36.3%), and α-pinene (1.8–21.4%) were identified as major constituents, alongside sesquiterpenes such as caryophyllene oxide [7]. These distinct variations in chemical profile are likely driven by differences in environmental conditions, geographical origin, plant phenological stages, and harvesting practices, all of which strongly influence secondary metabolite biosynthesis in aromatic plants [12,13,14]. This pattern suggests differential activation of biosynthetic pathways (methylerythritol phosphate (MEP) pathway and mevalonate (MVA) pathway, across the samples, resulting in distinct chemical profiles. Such compositional diversity is consistent with previous reports on *Senecio* species, which are known to exhibit considerable variability in essential oil composition [15,16].

**Figure 3:**
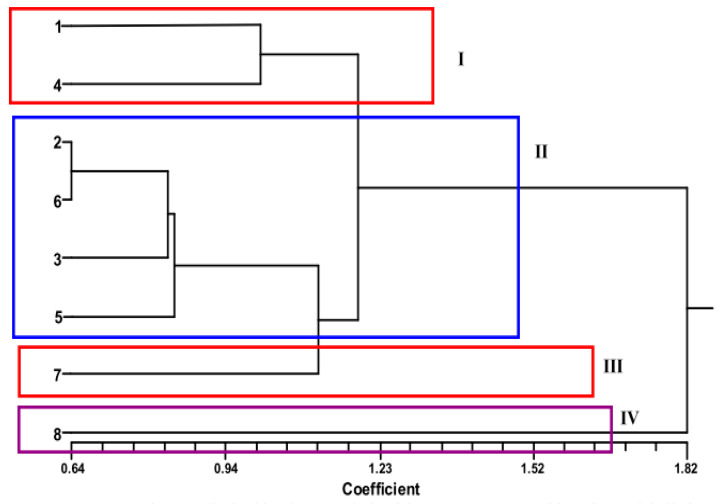
Dendrogram obtained from hierarchical cluster analysis of percentage composition of *S. polyanthemoides* essential oils based on unweighted pair-group method with arithmetic average (UPGMA). For abbreviations, see Table 1.

The results of the hierarchical cluster analysis further clarified the relationships among the samples by revealing four distinct clusters (Figure 3), thereby confirming the presence of pronounced chemical variability within the species. Cluster I comprised samples S1 and S4, collected from Empangeni and Gingindlovu, respectively. This cluster was characterized by relatively low concentrations of cis-(β)-ocimene (0.3–0.6%) and trans-(β)-ocimene (0.4–1.3%) when compared to the oils of other *S. polyanthemoides* samples, suggesting a reduced contribution of these monoterpene components to the overall profile. Cluster II included samples S2 (University of KwaZulu-Natal, Westville campus), S3 (Umhlanga), S5 (University of Zululand, KwaDlangezwa campus), and S6 (Mtunzini). This group exhibited high concentrations of α-pinene (8.9–15.0%), β-pinene (10.3–20.6%), myrcene (9.4–17.5%), and limonene (3.7–25.1%), with S2 and S6 showing particularly close similarity. Notably, this cluster was devoid of *p*-cymene, distinguishing it clearly from other groups. Cluster III consisted solely of sample S7 collected from Tongaat, which was unique in containing β-phellandrene (12.8%) as a significant constituent, indicating a distinct chemical profile. Cluster IV comprised sample S8 collected from Port Duruford and was characterized by high levels of *p*-cymene (10.6%), cis-β-ocimene (12.5%), and germacrene D (17.7%), but notably lacked α-pinene, a compound commonly present in other samples.

**Figure 4:**
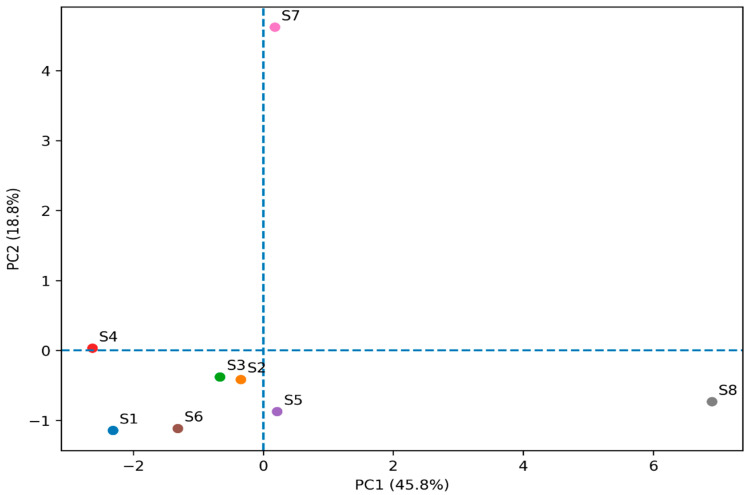
Two-dimensional (2D) PCA score plot (PC1 vs. PC2) showing the distribution of eight *S. polyanthemoides* populations based on their essential oil composition. The axes represent the principal components, with the percentage of total variance explained indicated in parentheses. Samples are grouped according to similarities in their chemical profiles.

These clustering patterns demonstrate that the essential oil composition of *S. polyanthemoides* varies not only quantitatively but also qualitatively, with certain compounds serving as distinguishing markers for specific groups. The separation of samples into these clusters highlights the role of key constituents such as α-pinene, β-pinene, myrcene, *p*-cymene, and germacrene D in driving chemical differentiation. In addition, the principal component analysis (PCA) generated from the compositional dataset (Figure 4) further corroborated the hierarchical clustering results by demonstrating substantial interpopulation variability among the essential oil samples. The two-dimensional PCA score plot revealed distinct grouping and clear separation of the populations according to their chemical profiles. Principal component 1 (PC1) accounted for 45.8% of the total variance, while principal component 2 (PC2) explained an additional 18.8%, giving a cumulative variance of 64.6% for the first two components. Samples S1–S6 clustered relatively close together, reflecting similarities in their monoterpene-dominated compositions, particularly with respect to α-pinene, β-pinene, myrcene, and limonene contents. In contrast, sample S8 was distinctly separated along PC1, largely due to its high levels of germacrene D (17.7%) and α-humulene (6.9%), whereas S7 showed clear separation along PC2, likely associated with elevated concentrations of β-phellandrene (12.8%) and α-terpinene (5.7%). The observed segregation pattern confirms that the chemical variability among the populations is systematic and biologically significant rather than random, suggesting the occurrence of marked compositional heterogeneity and possible chemotypic differentiation within the investigated populations of *S. polyanthemoides*.

In the present study, however, the clusters were derived from statistical analysis of samples collected from different locations at a specific time, and there is no evidence regarding the genetic basis or temporal stability of the observed chemical profiles. Therefore, although the clusters clearly indicate the presence of chemically distinct groups, they cannot be conclusively classified as true chemotypes. Rather, they are more appropriately described as putative chemotypes or chemotype-like chemical variants. This interpretation is consistent with previous studies on *Senecio* species, where essential oil composition has been shown to vary significantly in response to both genetic and environmental factors [15].

The variability observed in this study is in agreement with earlier investigations of *Senecio* species, which have reported fluctuations in major constituents such as β-caryophyllene, germacrene D, α-pinene, and limonene [17,18]. In particular, studies on *Senecio vulgaris* have demonstrated that essential oil composition can vary depending on ecological conditions, further supporting the role of environmental factors in shaping chemical profiles [15]. The presence or absence of specific compounds, such as β-phellandrene in Cluster III or *p*-cymene in Cluster IV, further emphasizes the extent of chemical heterogeneity within the species [7,10].

The observed differences in chemical composition have important implications for the biological activity of the essential oils [15,16]. Compounds such as β-caryophyllene, α-pinene, and caryophyllene oxide are known to possess significant pharmacological properties, including antimicrobial, anti-inflammatory, and antifungal activities. Variations in their relative abundance may therefore influence the therapeutic potential of the oils, suggesting that different clusters may exhibit different levels of biological activity. This highlights the importance of detailed chemical characterization in the evaluation and utilization of medicinal plants.

In addition to their pharmacological relevance, the results of this study have important chemotaxonomic implications. The presence and relative abundance of specific terpenoids may serve as useful markers for differentiating populations within *S. polyanthemoides*. The clustering patterns observed provide insight into the chemical diversity of the species and may contribute to future taxonomic and phylogenetic studies. Furthermore, the observed variability has practical significance for industrial applications, particularly in the selection of plant material for essential oil production. Understanding the factors that influence chemical composition is essential for ensuring consistency, quality control, and standardization of herbal products.

Overall, the application of multivariate statistical analysis, including hierarchical clustering and principal component analysis, proved to be a powerful tool for interpreting complex compositional data, enabling the identification of key compounds responsible for variability and the classification of samples into chemically meaningful groups. The results demonstrate that *S. polyanthemoides* exhibits substantial intraspecific chemical diversity, as reflected in the variation in essential oil composition and the clustering patterns observed. While these clusters suggest the presence of chemically distinct groups, they should be regarded as putative chemotypes rather than definitive chemotypes, pending further investigation. Future studies incorporating molecular characterization, seasonal variation, and environmental analysis will be necessary to confirm the existence of true chemotypes and to fully elucidate the factors governing chemical variation in this species.

### 2.2. Antibacterial Activity

#### 2.2.1. Agar Disc Diffusion

The antibacterial activity of *S. polyanthemoides* essential oils demonstrated inhibitory effects against the majority of the tested bacterial strains (Table 3). The oils exhibited strong activity against *Bacillus cereus*, *Bacillus pumilus*, *Staphylococcus aureus* strains, *Enterococcus faecalis*, and *Escherichia coli*, with zones of inhibition ranging from 8.0 ± 0.8 to 27.3 ± 0.9 mm. Moderate activity was observed against *Klebsiella pneumoniae*, *Proteus vulgaris* (ATCC 6538), *Pseudomonas aeruginosa*, and *Serratia marcescens*, with inhibition zones of 8.0 ± 1.0 to 17.7 ± 1.5 mm. The oils showed the least activity against *Proteus vulgaris* (CSIR 0030) and *Enterobacter cloacae*, with zones of inhibition ranging from 8.0 ± 0.0 to 9.0 ± 0.8 mm.

#### 2.2.2. Minimum Inhibitory Concentrations

The essential oils of *S. polyanthemoides* demonstrated variable inhibitory activity across the twelve bacterial strains tested (Table 4). MIC values ranged from 0.08 to 10.0 mg/mL, indicating a broad spectrum of efficacy. The oils exhibited the strongest inhibitory effects against *Bacillus pumilus*, *Enterococcus faecalis*, *Escherichia coli*, and *Staphylococcus aureus*, with MICs ≤ 5.0 mg/mL. Moderate inhibition was observed for *Bacillus cereus*, *Klebsiella pneumoniae*, *Proteus vulgaris* (ATCC 6538), *Pseudomonas aeruginosa*, and *Serratia marcescens*, whereas minimal or negligible activity was recorded for *Proteus vulgaris* (CSIR 0030) and *Enterobacter cloacae*. Among the individual samples, S1, S3, and S8 displayed the highest antibacterial potency, with MICs between 0.08 and 0.31 mg/mL against *B. pumilus*, *E. faecalis*, *E. coli*, and *S. aureus*. Notably, samples S1 and S8 were particularly effective against *S. aureus* strains. Comparison with standard antibiotics—chloramphenicol, gentamycin, and tetracycline—showed that the essential oils exhibited activities ranging from moderate to strong, with Gram-positive bacteria generally more susceptible than Gram-negative strains.

The antibacterial activity results, as indicated by the zones of inhibition and MIC values, were further supported by statistical analysis, where different superscript letters denote significant differences among the tested samples. Samples sharing the same letters were not significantly different (*p* > 0.05), indicating comparable levels of antibacterial efficacy, whereas those with different letters exhibited statistically distinct activities (*p* < 0.05). This statistical separation confirms that the observed variation in antibacterial performance among the essential oil samples is not random but reflects meaningful differences in their chemical composition. In particular, samples exhibiting larger zones of inhibition and lower MIC values, grouped under distinct significance categories, likely correspond to higher concentrations of active constituents or synergistic interactions among major and minor compounds.

These findings are consistent with previous studies demonstrating that the antimicrobial activity of essential oils is strongly influenced by their chemical composition and the relative abundance of bioactive constituents [19,20]. Essential oils have been widely reported to exhibit antibacterial effects against a broad range of pathogenic microorganisms, with variability in efficacy attributable to compositional differences and potential synergistic interactions among components [21,22,23]. However, while statistical significance confirms differences among samples, it does not directly identify the specific compounds responsible for the observed effects, underscoring the need for further bioassay-guided fractionation and compound-level characterization to establish structure–activity relationships [24].

### 2.3. Antioxidant Activity

#### 2.3.1. 2,2-Diphenyl-1-picrylhydrazyl (DPPH) Radical Scavenging Activity

The DPPH assay is a widely employed spectrophotometric method for evaluating the antioxidant capacity of plant-derived compounds, including essential oils [25]. It acts as a lipophilic model, mimicking radicals formed during lipid autoxidation to measure the scavenging power of antioxidants [26]. Table 5 shows the inhibitory concentrations (IC_50_) of *S. polyanthemoides* essential oils, BHA, BHT, Ascorbic acid and α-tocopherol on DPPH radicals scavenging activity. Although, the standard reference antioxidants (BHA, BHT, ascorbic acid and α-tocopherol) demonstrated higher inhibitory activity with IC_50_ ranging from below 10 to 10.70 µg/mL. When compared, the oil samples exhibited weak DPPH radical scavenging activity with IC_50_ values greater than 250 µg/mL, except sample S7 which showed slightly improved activity (IC_50_ = 219.32 µg/mL). The results shows that *S. polyanthemoides* essential oils possess limited hydrogen-donating capacity against DPPH radicals, which may be attributed to low concentrations of phenolic constituents typically responsible for strong radical scavenging activity [27]. Hence, the dominating effect of the oils by terpenoids rather than phenolic constituents, explain the weaker DPPH activity observed in this study, as terpenoids generally exhibit lower radical scavenging potency compared with phenolic antioxidants.

#### 2.3.2. Hydroxyl Radical Scavenging Activity

The IC_50_ values (Table 5) of the samples shows that many exhibited strong hydroxyl radical scavenging activity with scavenging activity (IC_50_) ranging from <10.00 to 50.87 µg/mL. While, the hydroxyl radical scavenging activities of the oil samples of S1, S5, S6, S7 and S8 were comparable to the commercial antioxidants (IC_50_ < 10.0 μg/mL). The results of strong hydroxyl radical scavenging observed in some oil samples may suggest the presence of compounds capable of metal chelation or reactive oxygen species neutralization, thus contributing to protective biological effects [28].

#### 2.3.3. Nitric Oxide Radical Scavenging Activity

The inhibitory concentrations (IC_50_) generated by the oil samples from nitric oxide radical are given in Table 5. The result shows moderate activity for some oil samples (S1, S4, S7 and S8), which may help in preventing the chains of reactions and diseases caused by excess nitric oxide radical generation such as inflammation, cancer and other physical or mental disorders that are harmful to human health [29].

#### 2.3.4. Reducing Power

Table 6 shows the reducing potential of *S. polyanthemoides* essential oils toward Fe^3+^, when assessed using the potassium ferricyanide reduction assay [11]. Among the tested oils, S5 and S7 exhibited notable reducing activity, comparable to the standard antioxidants BHA, BHT, ascorbic acid, and α-tocopherol. The overall order of reducing power was as follows: ascorbic acid > BHA > α-tocopherol > BHT > S5 > S7. The relatively high reducing activity of S5 and S7 can be attributed to their chemical profiles, particularly the presence of monoterpenes and oxygenated constituents, which are known to facilitate electron transfer and stabilize free radicals through redox mechanisms. These findings suggest that specific compositional features of *S. polyanthemoides* oils contribute to their antioxidant potential and may be exploited for applications in natural antioxidant formulations.

### 2.4. Brine Shrimp Lethality Assay

The brine shrimp lethality assay of *S. polyanthemoides* essential oils was evaluated using *Artemia salina*. This assay provides a rapid, cost-effective, and reliable method for preliminary toxicity screening of natural products and has been shown to correlate with cytotoxic and antitumor activities [30]. The lethality effects of the essential oils after 24 h of exposure are presented in Table 7. No mortality was observed in the negative controls (artificial seawater and distilled water), confirming the validity of the assay. However, the solvent control (1% DMSO) exhibited low but measurable toxicity, with an LC_50_ value of 59.70 µg/mL (95% CI: 29.62–130.86). This level of toxicity is substantially lower than that of the essential oils and is considered negligible in comparison.

The essential oils demonstrated concentration-dependent lethality, with increasing concentrations resulting in higher mortality of *A. salina* nauplii. The 24 h LC_50_ values ranged from 9.63 to 16.52 µg/mL, indicating significant cytotoxic activity. Among the samples, S6 exhibited the highest toxicity (LC_50_ = 9.63 µg/mL), while S3 showed comparatively lower activity (LC_50_ = 16.52 µg/mL). A similar trend was observed for LC_90_ values. To the best of our knowledge, there are no previous reports on the cytotoxicity of *S. polyanthemoides* essential oils using the brine shrimp lethality model. However, the results are consistent with studies on other plant essential oils [31]. The relatively low LC_50_ values suggest the presence of potent bioactive constituents, warranting further investigation.

### 2.5. Larvicidal Activity

The larvicidal activity of *Senecio* species essential oils is presented in Table 8. The oils exhibited considerable variation in toxicity against fourth-instar larvae, with LC_50_ values ranging from 30.70 to 50.70 µg/mL, indicating moderate to strong larvicidal potential depending on the sample. Among the tested oils, S5 showed the highest activity (LC_50_ = 30.70 µg/mL), followed by S8 (33.70 µg/mL) and S7 (36.31 µg/mL), whereas S1 displayed the lowest activity (50.70 µg/mL). These findings suggest that the bioactivity is highly dependent on chemical composition and relative abundance of active constituents, which is consistent with previous reports on plant-derived essential oils used in mosquito control strategies [32,33,34]. The LC_90_ values further supported these trends, with S7 exhibiting the lowest LC_90_ (118.84 µg/mL), indicating superior efficacy at higher mortality thresholds. This suggests that S7 maintains stronger lethality even at elevated survival resistance levels, making it a promising candidate for vector control applications. In contrast, S1 required the highest concentration to achieve 90% mortality (170.69 µg/mL), reflecting comparatively weaker efficacy. Slope values ranged from 1.88 to 2.41, indicating variability in the dose–response relationship among samples. S7 exhibited the steepest slope (2.41 ± 0.32), suggesting a more homogeneous larval response to increasing concentrations, whereas S1 showed a flatter slope (1.88 ± 0.33), indicating greater heterogeneity in susceptibility. The χ^2^ values (1.12–1.89) were low across all treatments, confirming a good fit of the probit model and the reliability of LC estimates.

Potency grouping further highlighted statistically significant differences among treatments. S7 (group “a”) demonstrated the highest larvicidal potency, followed by S5 and S8 (group “ab”), while S1 (group “d”) was the least active. Intermediate activity was observed for S2, S3, S4, and S6, suggesting moderate effectiveness. The absence of larvicidal activity in both controls (1% Dimethyl sulfoxide and distilled water), confirmed that mortality was solely attributable to the bioactive constituents of the essential oils. The observed larvicidal activity is likely associated with the presence of bioactive terpenoids and oxygenated monoterpenes commonly reported in *Senecio* essential oils, which are known to disrupt insect neurological and respiratory systems [33,35,36,37]. Similar mechanisms have been documented for plant-derived essential oils exhibiting neurotoxic and membrane-disruptive effects on mosquito larvae, leading to rapid mortality and growth inhibition [34,35,36,37,38,39]. Overall, the results suggest that *Senecio* essential oils possess promising larvicidal properties and may serve as potential botanical alternatives for mosquito vector management.

## 3. Material and Methods

### 3.1. Chemicals/Media/Reagents

All microbiological media, analytical-grade chemicals, reagents, and reference standards were sourced from certified global distributors. Mueller-Hinton Agar No. 2 (pH 7.4) and Mueller-Hinton Broth (pH 7.4) were manufactured by Oxoid Laboratories (Thermo Fisher Scientific, Hertfordshire, UK) and supplied via Merck (Sigma-Aldrich, Modderfontein, Johannesburg, South Africa). Antibiotic discs including. Gentamycin (25 µg), Chloramphenicol (25 µg) and Tetracycline (30 µg), as well as, 2,2-diphenyl-1-picrylhydrazyl (DPPH), trichloroacetic acid (TCA), thiobarbituric acid (TBA), Griess Illosvoy reagents (naphthylethylenediamine dihydrochloride and sodium nitroprusside), sulphanilic acid reagent (0.33% in 20% glacial acetic acid), assay salts, and antioxidant standards (butylated hydroxyanisole [BHA], butylated hydroxytoluene [BHT], ascorbic acid, and α-tocopherol) were purchased from Merck (Sigma-Aldrich, Modderfontein, Johannesburg South Africa). Additionally, *Artemia salina* cysts (brine shrimp eggs) were obtained from Fish Designs (Distributor of Live Ornamental Fish and Products), Mtunzini, South Africa.

### 3.2. Plant Materials

**Figure 5:**
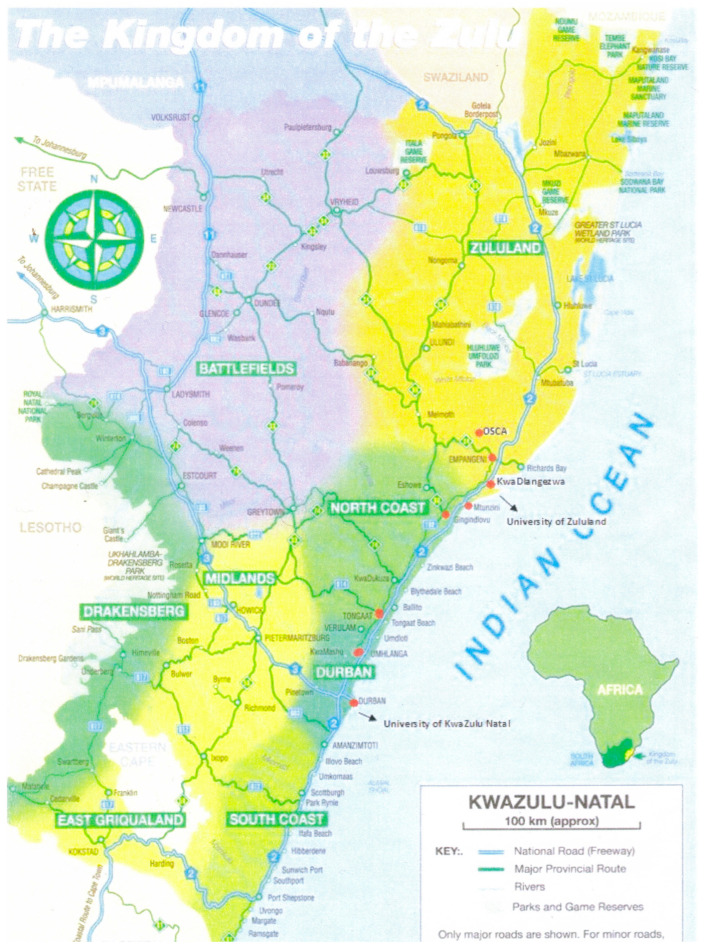
The map of the North Coast Part of KwaZulu-Natal Province, South Africa. The red points correspond to locations where plant samples were collected (Table 1).

Fresh plant materials of *S. polyanthemoides* growing wild in different district municipalities of KwaZulu-Natal Province, South Africa (Figure 5), were randomly collected from King Cetshwayo (S1, S4, S5 and S6) and iLembe (S8) district municipalities, and eThekwini Metropolitan Municipality (S2, S3 and S7), where 5–8 individual plants were collected from each population at the same time as homogeneous samples of the population) at flowering stage. Taxonomical identification of the plant materials were confirmed by Dr. S. J. Siebert and Dr. N.R. Ntuli. Voucher specimens (Table 1) had been deposited at the University of Zululand, Herbarium.

### 3.3. Hydrodistillation of Essential Oils

Aerial parts (above the ground organs consisting of the leaves, stems and flowers) of *S. polyanthemoides* (500 g) collected from each location were subjected to hydrodistillation using a Clevenger-type apparatus for 3 h, following the British Pharmacopoeia guidelines [40]. The volatile oils, distilled over water, were collected in the receiver arm of the apparatus and transferred into clean, pre-weighed sample bottles. The samples were stored under refrigeration until further chemical and biological analyses.

### 3.4. Chemical Analyses of Essential Oils

To ensure robust chemical characterization, Gas Chromatography with Flame Ionization Detection (GC-FID) was utilized strictly for quantitative analysis (peak area percentages), while Gas Chromatography-Mass Spectrometry (GC-MS) was employed independently for qualitative compound identification via mass spectral matching and Retention Indices (RI).

#### 3.4.1. Gas Chromatography (GC-FID Quantification)

Quantitative analyses of the essential oils were performed using a Hewlett-Packard HP 6820 gas chromatograph equipped with a DB-5 capillary column (60 m × 0.25 mm i.d., film thickness 0.25 µm) and a flame ionization detector (FID) (Agilent Technologies, Santa Clara, California, United States). The injector split ratio was set to 1:25. The oven temperature program was initiated at 50 °C (held for 2 min), increased to 240 °C at a heating rate of 5 °C/min, and the final temperature was maintained for 10 min. The injection and detector temperatures were maintained at 200 °C and 240 °C, respectively. Hydrogen served as the carrier gas. An aliquot of 0.5 μL of the essential oil diluted in *n*-hexane (1:100, *v*/*v*) was injected. Relative percentages of the volatile constituents were calculated by electronic integration of the GC-FID peak areas without the use of correction factors.

#### 3.4.2. Gas Chromatography-Mass Spectrometry

The chemical composition of the oils was determined using a Hewlett–Packard GC system (HP 6890) coupled to a Hewlett–Packard 5973 mass selective detector (GC–MS) (Agilent Technologies, Santa Clara, California, United States). Separation was carried out on an HP-5MS fused-silica capillary column (30 m × 0.25 mm i.d.; film thickness 0.25 µm). The oven temperature was programmed from 70 °C to 240 °C at a rate of 5 °C/min. The mass spectrometer was operated in electron ionization (EI) mode at 70 eV, with the ion source temperature maintained at 240 °C. Helium served as the carrier gas at a constant flow rate of 1.0 mL/min. Mass spectra were recorded over the range of 35–425 amu. A 1.0 µL aliquot of the essential oil, diluted in hexane, was injected into the GC–MS system for analysis. Retention indices (RIs) were calculated using a homologous series of *n*-alkanes analyzed under the same chromatographic conditions.

#### 3.4.3. Identification of Essential Oil Constituents

Essential oil components were identified based on their retention relative indices (RRIs), which were determined by co-injection with a homologous series of *n*-alkanes under identical GC–MS conditions. Further confirmation of individual compounds was achieved by comparing their mass spectra with those from an in-house MS library constructed from authentic standards and previously characterized essential oil components. Additionally, the retention indices were compared with reported literature values to ensure accurate identification [41,42,43].

### 3.5. Multivariate Analysis

#### 3.5.1. Hierarchical Cluster Analysis (HCA)

Eight *S. polyanthemoides* essential oil samples were analysed as operational taxonomic units (OTUs) to evaluate chemical diversity and identify potential chemotypes. The relative percentage composition of 18 major constituents—α-pinene, sabinene, β-pinene, myrcene, α-phellandrene, α-terpinene, *p*-cymene, β-phellandrene, limonene, *cis*-β-ocimene, *trans*-β-ocimene, γ-terpinene, β-caryophyllene, α-humulene, germacrene D, bicyclogermacrene, and δ-cadinene—was determined by standard gas chromatography methods. These compositional data were subsequently used to assess chemical relationships among the samples. Hierarchical cluster analysis (HCA) was performed using NTSYSpc software, version 2.2 to classify the samples according to their chemical profiles [44,45]. The percentage composition data were arranged in a sample × constituent matrix, and similarity among samples was estimated using Pearson correlation coefficients. Cluster formation was carried out using the unweighted pair-group method with arithmetic mean (UPGMA) within the Sequential Agglomerative Hierarchical Nested (SAHN) routine of the software. The resulting dendrogram was used to visualize chemotypic relationships among samples. Cluster boundaries were defined according to natural separations observed in the similarity coefficients, enabling classification of the oils into distinct groups with related chemical compositions. This approach facilitated the identification of potential chemotypes within *S. polyanthemoides*.

#### 3.5.2. Principal Component Analysis

Principal Component Analysis (PCA) was employed to evaluate compositional variation among the eight populations of *S. polyanthemoides* based on their essential oil constituents [46]. Prior to analysis, the data matrix consisting of the relative percentages of the identified volatile compounds was standardized to reduce the influence of differences in variable scale and to ensure equal contribution of all variables to the analysis. The analysis was performed using the covariance matrix, and principal components with eigenvalues greater than 1.0 were considered significant according to the Kaiser criterion. A two-dimensional (2D) PCA score plot based on principal component 1 (PC1) and principal component 2 (PC2) was generated to visualize the distribution, clustering, and relationships among the essential oil samples. The percentage variance explained by each principal component was indicated on the corresponding axes, while the variables contributing most significantly to sample discrimination were identified from the component loadings. The application of PCA as a chemometric tool for interpreting complex phytochemical datasets has been widely reported in essential oil and metabolomic studies.

### 3.6. Antibacterial Activity

#### 3.6.1. Microbial Strains

Twelve reference bacterial strains obtained from Applied and Environmental Microbiology Research Group (AEMREG), Department of Biochemistry and Microbiology, University of Fort Hare, Alice, South Africa were used to determine the antibacterial activity of *S. polyanthemoides* essential oils. The test panel comprised Gram-positive bacteria: *Bacillus cereus* (ATCC 10702), *Bacillus pumilus* (ATCC 14884), *Staphylococcus aureus* (ATCC 3983), *Staphylococcus aureus* (ATCC 6538), and *Enterococcus faecalis* (ATCC 29212), as well as Gram-negative bacteria: *Enterobacter cloacae* (ATCC 13047), *Escherichia coli* (ATCC 4983), *Klebsiella pneumoniae* (ATCC 2983), *Proteus vulgaris* (ATCC 6830), *Proteus vulgaris* (CSIR 0030), *Pseudomonas aeruginosa* (ATCC 19582), and *Serratia marcescens* (ATCC 9986). The stock cultures of the bacterial strains were maintained at 4 °C on Mueller-Hinton agar (Oxoid, UK) until use. Prior to experimental assays, fresh cultures were prepared to ensure viability and consistency.

#### 3.6.2. Agar Disc Diffusion Assay

The antibacterial potential of *S. polyanthemoides* essential oils was evaluated using the agar disk diffusion assay, following standard protocols [47]. Bacterial cultures were prepared (overnight) in 20 mL of Mueller-Hinton broth (MHB) (Oxoid, Wesel, Germany) and incubated at 37 °C. The turbidity of each culture was adjusted with sterile saline to match that of a McFarland no. 5 standard, corresponding to approximately 1.0 × 10^8^ CFU/mL. Sterile Petri dishes (90 mm, Merck, Darmstadt, Germany) containing 12 mL of molten Mueller-Hinton agar were inoculated by evenly spreading 100 µL of the standardized bacterial suspension over the agar surface. Sterile Whatman No. 1 filter paper discs (6 mm diameter) were then aseptically placed on the surface of the inoculated plates. Each disc was impregnated with 10 µL of essential oil dissolved in 1% dimethyl sulfoxide (DMSO). The plates were incubated at 37 °C for 24 h to allow microbial growth and interaction with the oils. After incubation, the diameters of the inhibition zones surrounding each disc were measured in millimeters using a ruler. Each experiment was performed in triplicate to ensure reproducibility. Gentamycin (25 µg), Chloramphenicol (25 µg) and Tetracycline (30 µg), and 1% DMSO were included as positive and negative controls, respectively. The antibacterial activity was interpreted based on the size of the inhibition zones, with larger zones indicating stronger antimicrobial efficacy.

#### 3.6.3. Minimum Inhibitory Concentration

The minimum inhibitory concentration (MIC) of *S. polyanthemoides* essential oils was determined using the 96-well microtiter dilution method as described previously [48]. Overnight bacterial cultures were grown in Müller-Hinton (MH) broth at 37 °C, and a 1:1 dilution of each culture in fresh MH broth was prepared prior to the assay. Serial two-fold dilutions of the essential oils were made to achieve concentrations ranging from 10 mg/mL to 0.078 mg/mL. Aliquots of 100 µL of bacterial suspension, with an approximate inoculum of 1.0 × 10^8^ CFU/mL, were added to each well containing the diluted essential oils. The plates were incubated at 37 °C for 24 h. After incubation, 40 µL of 0.2 mg/mL p-iodo-nitrotetrazolium violet (INT) solution was added to each well, and the plates were further incubated at 37 °C for 30–60 min. The MIC was defined as the lowest concentration of essential oil that produced near-complete inhibition of visible microbial growth. Gentamycin (25 µg), Chloramphenicol (25 µg), and Tetracycline (30 µg), as a standard positive reference antibiotics. While, 1% dimethyl sulfoxide (DMSO) as the negative solvent control.

### 3.7. Antioxidant Activity

#### 3.7.1. 2,2-Diphenyl-1-picrylhydrazyl (DPPH) Radical Scavenging Activity

The free radical scavenging activity of *S. polyanthemoides* essential oils was assessed using the 2,2-diphenyl-1-picrylhydrazyl (DPPH) assay as described previously [18]. Briefly, 0.2 mL of essential oil solutions at concentrations ranging from 10 to 250 µg/mL in methanol were added to 2.7 mL of 1.0 × 10^−4^ M DPPH solution prepared in methanol. The reaction mixtures were incubated in the dark for 60 min to prevent light-induced degradation. After incubation, the absorbance was measured at 517 nm using a Genesys 20 UV–Visible spectrophotometer.

DPPH scavenging activity (%): = {1 − (S − SB)/(C − CB)} × 100

where S is the absorbance of the sample, SB is the absorbance of the sample blank (2 mL methanol + 0.2 mL essential oil), C is the absorbance of the control (2 mL DPPH solution + 0.2 mL methanol), and CB is the absorbance of the control blank (methanol). The concentration of essential oil required to scavenge 50% of DPPH radicals (IC_50_) was determined from a plot of percentage inhibition versus oil concentration.

#### 3.7.2. Nitric Oxide Radical Scavenging Activity

The scavenging potential of nitric oxide from *S. polyanthemoides* essential oils was assessed using the Griess–Illosvoy reaction as described previously [49]. This method is based on the generation of nitric oxide from sodium nitroprusside in an aqueous medium at physiological pH, followed by its oxidation to nitrite ions, which are subsequently detected colorimetrically. Briefly, a total reaction volume of 3 mL was prepared, consisting of 2 mL of 10 mM sodium nitroprusside, 0.5 mL of 0.01 M phosphate-buffered saline (pH 7.4), and 0.5 mL of essential oil solutions at different concentrations. The reaction mixtures were incubated at 25 °C for 150 min to allow nitric oxide formation. After incubation, 0.5 mL of the reaction mixture was combined with 1 mL of sulphanilic acid reagent (0.33% w/v in 20% glacial acetic acid) and left to stand for 5 min to complete the diazotization process. Subsequently, 1 mL of 0.1% (*w*/*v*) naphthylethylenediamine dihydrochloride was added, and the mixture was allowed to react for 30 min under diffused light, leading to the formation of a pink azo dye.

#### 3.7.3. Hydroxyl Radical Scavenging Activity

The hydroxyl radical scavenging activity of *S. polyanthemoides* essential oils was evaluated using the Fenton reaction method as previously described [50]. The reaction mixture comprised 450 µL of 0.2 M phosphate buffer (pH 7.0), 150 µL of 10 mM 2-deoxyribose, 150 µL of 10 mM FeSO_4_–EDTA, 525 µL of distilled water, and 75 µL of the essential oil solution at varying concentrations was initiated by the addition of 150 µL of 10 mM H_2_O_2_ and incubated at 37 °C for 4 h. Following incubation, the reaction was terminated by adding 750 µL of 2.8% (*w*/*v*) trichloroacetic acid (TCA) and 750 µL of 1% (*w*/*v*) thiobarbituric acid (TBA). The mixture was heated in a boiling water bath for 10 min, then allowed to cool to room temperature. The absorbance of the resulting solution was measured at 532 nm using a Genesys 20 UV–Visible spectrophotometer.

#### 3.7.4. Percentage Inhibition Scavenging Activity

Butylated hydroxyanisole (BHA), butylated hydroxytoluene (BHT), ascorbic acid (AA), and α-tocopherol were used as standard reference antioxidants. All assays were performed in triplicate, and results are expressed as mean ± standard error of the mean (SEM). The inhibitory effects of *S. polyanthemoides* essential oils on nitric oxide and hydroxyl radical scavenging activities were calculated using the following equation:

% Inhibition = {(A_0_ − A_1_)/A_0_ × 100}

where A_0_ is the absorbance value of the fully oxidized control and A_1_ is the absorbance of the extract. The inhibitory concentration providing 50% inhibition (IC_50_) was calculated from the graph of percentage inhibition against *S. polyanthemoides* essential oils concentrations.

#### 3.7.5. Reducing Power Assay

The essential oils were assessed according to the previously described method [51]. In brief, 2.5 mL of essential oil solutions at concentrations at 10 to 250 µg/mL in methanol were combined with 2.5 mL of 0.2 M phosphate buffer (pH 6.6) and 2.5 mL of 1% (*w*/*v*) potassium ferricyanide [K_3_Fe(CN)_6_]. The mixture was incubated at 50 °C for 20 min to facilitate reduction. After incubation, 2.5 mL of 10% (*w*/*v*) trichloroacetic acid was added to terminate the reaction, and the mixture was centrifuged at 1000 rpm for 10 min. Thereafter, 2.5 mL of the supernatant was mixed with an equal volume of distilled water and 0.5 mL of 0.1% (*w*/*v*) ferric chloride (FeCl_3_). The absorbance of the resulting solution was measured at 700 nm using a Genesys 20 UV–Visible spectrophotometer. Standard antioxidants, including ascorbic acid, butylated hydroxyanisole (BHA), butylated hydroxytoluene (BHT), and α-tocopherol, served as positive controls. Increased absorbance values were interpreted as indicative of stronger reducing power.

### 3.8. Brine Shrimp Lethality Assay

Nauplii of *Artemia salina* were employed for the brine shrimp lethality assay following established procedures [52]. In brief, ten larvae were exposed to 50 µL of essential oil solutions at concentrations ranging from 10 to 250 µg/mL, prepared in 1% dimethyl sulfoxide (DMSO) and adjusted to a final volume of 5 mL with artificial seawater. A negative control containing only artificial seawater and a solvent control consisting of 1% DMSO in artificial seawater without essential oil were included concurrently. After 24 h of incubation under illumination at room temperature, nauplii were examined and considered dead when they showed no movement after gentle probing. The percentage mortality was calculated and corrected using Abbott’s formula where necessary to account for control mortality. LC_50_ and LC_90_ values were determined using probit analysis (Probit Analysis Program, version 1.5). Results were expressed as LC_50_ and LC_90_ values with corresponding 95% confidence intervals, representing the concentrations (µg/mL) required to induce 50% and 90% mortality, respectively, after 24 h of exposure. The assay was used as a preliminary toxicity screening of the essential oil.

### 3.9. Larvicidal Activity

Larvicidal bioassays against Culex quinquefasciatus were conducted following the standard procedure described by the World Health Organization for mosquito larval susceptibility testing, with slight modifications [53]. Briefly, five fourth-instar larvae were collected using a Pasteur pipette, gently blotted on filter paper to remove excess water, and transferred with a fine brush into 100 mL beakers containing 29.0 mL of degassed distilled water and 1000 µL of test solution at varying concentrations. The contents were gently agitated to ensure uniform dispersion. Each treatment was performed in triplicate and maintained at 25 ± 2 °C under a 12:12 h light–dark photoperiod. Two control groups were included and maintained under identical conditions: distilled water served as the negative control, while 1% Dimethyl sulfoxide (DMSO) in distilled water was used as the solvent control. Larval mortality was recorded after 24 h, and larvae were considered dead if they showed no movement upon probing with a fine needle. Percentage mortality was calculated and, where necessary, corrected using Abbott’s formula [54] to account for control mortality. The median lethal concentration (LC_50_) values were determined by probit analysis using the U.S. Environmental Protection Agency Probit Analysis Program (Version 1.5) [55]. Larvicidal activity was expressed as LC_50_ values (µg/mL) with corresponding 95% confidence intervals, representing the concentration required to achieve 50% larval mortality within 24 h.

### 3.10. Statistical Analysis

Statistical analyses were carried out using Microsoft Excel 2003, while IC_50_ values were computed with Origin 6.0. Comparisons among groups were performed using one-way analysis of variance (ANOVA) to assess statistical differences between treatments. A probability value (*p*) ≤ 0.05 was considered statistically significant, whereas *p* ≤ 0.01 was regarded as highly significant. Median lethal concentration (LC_50_), defined as the concentration required to cause 50% mortality, was determined by probit analysis using U.S. Environmental Protection Agency (EPA) Probit Analysis Program (Version 1.5).

## 4. Conclusions

This study demonstrates pronounced and previously underappreciated chemical variability in the essential oils of *Senecio polyanthemoides*, as revealed by GC–MS profiling combined with multivariate statistical analyses (HCA and PCA). The observed differences in the relative abundance of major constituents confirm substantial intraspecific diversity, consistent with prior reports describing compositional variability within the genus *Senecio* [19,20]. The strong concordance between HCA and PCA supports the robustness of the clustering patterns, indicating that the observed chemical differentiation reflects intrinsic biological variability rather than analytical or methodological artefacts. Such variability is in agreement with established evidence that essential oil composition is influenced by both genetic factors and environmental conditions [20,24]. However, the present dataset is insufficient to support definitive chemotype classification. Any chemotypic assignment remains preliminary and should be interpreted with caution. A more comprehensive evaluation incorporating a larger number of populations across different geographic regions, seasons, and environmental conditions is required to reliably distinguish genetic effects from ecological influences and to establish stable chemotypes [24].

A noteworthy finding of this study is the identification of limonene as a predominant constituent in several samples. Although limonene is a common monoterpene reported in many aromatic plant species and is associated with various ecological and biological functions, its prominence as a major component in *Senecio* species has not been previously documented. This suggests its potential value as a chemotaxonomic marker for *Senecio* taxa, particularly within South African populations. The in vitro biological activities observed further indicate the pharmacological potential of the essential oils. Nevertheless, the specific compounds responsible for these activities cannot be conclusively identified based on the current data. Future investigations should therefore employ bioassay-guided fractionation alongside advanced spectroscopic and chromatographic techniques to isolate and characterize the active constituents and to establish structure–activity relationships underlying the observed bioactivity.

## Figures and Tables

**Table 1:** Collection details, yield (% *w*/*w*), and physicochemical properties of *S. polyanthemoides* essential oils.

Sample Code	% Yield (*w*/*w*)	Voucher Specimen	Latitude /Longitude	Colour	Odour	Refractive Index
S1	0.10	LAWAL, OA 24 (ZULU)	28°44′00″ S /31°53′24″ E	Pale yellow	Herbaceous	1.4875
S2	0.12	LAWAL, OA 34 (ZULU)	29°52′58.8″ S /31°03′00″ E	Pale yellow	Herbaceous	1.4966
S3	0.10	LAWAL, OA 26 (ZULU)	29°43′54.1″ S /31°04′37.9″ E	Golden yellow	Herbaceous	1.5160
S4	0.11	LAWAL, OA 27 (ZULU)	29°01′46″ S /31°34′55″ E	Golden brown	Herbaceous	1.5875
S5	0.13	LAWAL, OA 23 (ZULU)	28°51′16″ S /31°50′45″ E	Pale yellow	Herbaceous	1.5292
S6	0.11	LAWAL, OA 28 (ZULU)	28°57′24″ S /31°45′15″ E	Pale yellow	Herbaceous	1.5080
S7	0.12	LAWAL, OA 29 (ZULU)	29°33′39″ S /31°07′20″ E	Pale yellow	Herbaceous	1.4983
S8	0.10	LAWAL, OA 32 (ZULU)	28°53′32″ S /31°50′40″ E	Pale yellow	Herbaceous	1.4772

S1—Empangeni, S2—Durban, S3—Umhlanga, S4—Gingindlovu, S5—KwaDlangezwa, S6—Mtunzini, S7—Tongaat, S8—Port Duruford.

**Table 2:** Chemical constituents identified in the essential oils of *S. polyanthemoides* by GC–MS analysis.

Compound ^a^	RI ^b^	RI ^c^	M^+^	Fi	IM	Percentage Composition							
						S1	S2	S3	S4	S5	S6	S7	S8
α-pinene	935	937	136	93, 91, 77	RI, MS	22.3	12.8	14.4	11.8	8.9	15.0	10.6	-
sabinene	947	946	136	93, 91, 69	RI, MS	-	4.9	-	-	-	-	-	-
β-pinene	969	968	136	93, 91, 77	RI, MS	20.0	10.3	20.6	17.3	12.9	14.8	15.8	0.5
myrcene	987	989	136	93, 69, 41	RI, MS	12.3	14.2	9.4	10.7	13.4	17.5	7.1	5.8
α-phellandrene	1020	1019	136	93, 79, 77	RI, MS	0.9	8.0	8.0	1.2	9.4	4.3	6.1	1.3
α-terpinene	1018	1016	136	93, 121, 79	RI, MS	t	0.1	-	0.8	1.3	0.8	5.7	-
*p*-cymene	1022	1022	134	119, 91, 77	RI, MS	13.0	-	12.2	-	15.3	-	-	10.6
limonene	1030	1031	136	68, 93, 121	RI, MS	11.7	15.8	3.7	33.6	8.1	25.1	-	6.1
β-phellandrene	1033	1033	136	93, 79, 77	RI, MS	-	-	-	-	-	-	12.8	-
*cis*-β-ocimene	1037	1037	136	93, 121, 69	RI, MS	0.6	8.7	7.7	0.3	7.8	5.8	2.7	12.5
*trans*-β-ocimene	1042	1044	136	93, 121, 69	RI, MS	1.3	7.1	5.8	0.4	7.6	7.4	5.2	6.3
γ-terpinene	1071	1068	136	93, 121, 77	RI, MS	0.6	1.5	1.0	1.6	0.6	0.9	1.8	-
*cis*-sabinene hydrate	1073	1076	154	71, 93, 59	RI, MS	-	-	0.2	-	-	-	0.1	-
α-terpinolene	1079	1078	136	93, 121, 79	RI, MS	t	0.6	0.5	0.5	-	-	1.1	-
α-naginatene	1085	1082	204	161, 105, 91	RI, MS	-	0.2	-	-	-	0.2	-	0.1
*trans*-sabinene hydrate	1093	1094	154	71, 93, 59	RI, MS	-	-	-	0.2	-	-	-	-
perillene	1099	1096	150	67, 95, 108	RI, MS	0.9	0.3	0.1	0.9	0.6	-	0.2	-
nonanal	1100	1102	142	57, 70, 41	RI, MS	-	0.1	t	-	-	-	0.1	-
α-campholene aldehyde	1105	1105	152	95, 81, 67	RI, MS	0.5	-	-	-	-	-	-	-
allo-ocimene	1109	1109	136	93, 121, 79	RI, MS	0.4	1.6	0.6	-	0.5	-	0.5	0.7
*cis*-*p*-mentha-2-en-l-ol	1131	1129	154	59, 93, 121	RI, MS	-	-	-	-	-	-	0.8	-
*trans*-*p*-mentha-2-en-l-ol	1137	1136	154	59, 93, 121	RI, MS	-	-	0.1	-	-	-	-	-
*trans*-pinocarveol	1145	1143	154	71, 93, 59	RI, MS	1.8	-	0.5	0.5	0.5	-	-	-
pinocarvone	1159	1160	152	95, 82, 108	RI, MS	-	t	0.1	0.3	0.3	-	0.2	-
*p*-mentha-1,5-dien-1,8-ol	1162	1163	154	59, 93, 121	RI, MS	0.4	-	-	-	-	-	0.2	-
terpinen-4-ol	1179	1177	154	71, 93, 59	RI, MS	1.3	2.0	2.5	2.8	1.4	-	4.9	2.0
cryptone	1181	1181	152	95, 67, 41	RI, MS	-	-	0.1	-	-	-	0.4	-
α-terpineol	1191	1189	154	59, 93, 121	RI, MS	0.4	0.4	0.6	0.2	0.6	-	1.3	0.4
myrtenal	1205	1202	150	95, 67, 41	RI, MS	1.0	0.1	0.2	0.4	0.5	-	0.6	-
3-nonenyl acetate	1225	1221	198	43, 61, 82	RI, MS	0.5	-	-	-	0.3	-	-	-
carvone	1248	1248	150	82, 95, 108	RI, MS	-	t	-	0.3	0.3	-	t	-
dihydroedulan I	1286	1284	222	161, 105, 91	RI, MS	-	-	0.2	-	-	-	0.3	-
bicycloelemene	1326	1327	204	161, 119, 93	RI, MS	-	-	-	-	-	-	0.1	-
α-cubebene	1348	1347	204	161, 105, 91	RI, MS	-	0.3	t	-	0.6	-	-	-
eugenol	1357	1359	164	149, 137, 109	RI, MS	-	-	-	-	-	-	0.2	-
α-copaene	1369	1367	204	161, 119, 93	RI, MS	0.3	-	0.1	t	0.6	-	-	-
*trans*-β-damascenone	1373	1376	190	121, 91, 69	RI, MS	-	-	-	-	-	-	0.1	-
*cis*-β-damascenone	1385	1381	190	121, 91, 69	RI, MS	-	-	-	-	t	-	-	0.3
β-elemene	1386	1386	204	161, 93, 105	RI, MS	t	0.3	0.2	t	0.4	-	1.0	1.5
β-cubebene	1391	1389	204	161, 105, 91	RI, MS	0.3	-	-	-	-	-	-	-
β-caryophyllene	1424	1422	204	133, 161, 93	RI, MS	0.3	1.1	0.3	0.4	0.8	1.0	1.1	3.5
α-humulene	1454	1454	204	161, 93, 105	RI, MS	0.9	0.9	0.5	0.4	1.6	1.7	1.6	6.9
*allo*-aromadendrene	1467	1469	204	161, 105, 91	RI, MS	-	0.1	t	-	-	-	0.1	-
germacrene D	1482	1478	204	161, 93, 105	RI, MS	1.7	3.2	1.4	1.0	3.6	2.8	4.0	17.7
bicyclogermacrene	1490	1489	204	161, 93, 119	RI, MS	-	0.7	0.2	-	0.2	-	1.3	2.0
α-muurolene	1495	1496	204	161, 105, 91	RI, MS	-	t	0.2	-	0.2	-	-	1.1
α-amophene	1499	1499	204	161, 105, 91	RI, MS	-	-	-	-	-	-	0.2	0.5
γ-cadinene	1512	1510	204	161, 105, 119	RI, MS	-	-	0.2	-	-	-	-	-
δ-cadinene	1517	1519	204	161, 105, 119	RI, MS	t	0.4	1.1	t	0.3	-	0.9	2.7
α-cadinene	1526	1529	204	161, 105, 119	RI, MS	-	-	-	-	-	-	-	0.1
*cis*-nerolidol	1532	1532	222	69, 93, 121	RI, MS	-	-	0.1	-	-	-	0.2	0.2
palustrol	1546	1548	222	69, 93, 121	RI, MS	-	-	-	-	-	-	0.1	-
spathulenol	1577	1571	220	59, 79, 161	RI, MS	0.3	0.2	0.2	0.7	0.2	-	0.5	1.1
caryophyllene oxide	1583	1583	220	91, 105, 161	RI, MS	0.7	0.2	0.1	0.6	0.2	-	0.3	-
globulol	1586	1585	222	69, 93, 121	RI, MS	-	-	0.2	0.3	-	-	-	-
viridiflorol	1591	1591	222	69, 93, 121	RI, MS	-	-		-	-	-	0.3	0.1
humulene epoxide	1602	1600	220	93, 107, 161	RI, MS	0.8	0.1	0.1	0.7	0.2	-	0.2	0.6
junenol	1611	1609	222	69, 93, 121	RI, MS	-	-	-	-	-	-	0.4	-
τ-cadinol	1634	1635	222	161, 105, 119	RI, MS	-	-	-	-	-	-	0.4	-
τ-muurolol	1638	1639	222	161, 105, 119	RI, MS	-	0.8	0.9	-	-	-	1.0	-
α-cadinol	1650	1651	222	161, 105, 119	RI, MS	-	-	-	-	-	-	1.4	0.1
1,6-germacradien-5-ol	1656	1658	222	69, 93, 121	RI, MS	-	-	0.2	-	-	-	-	-
mintisulfide	1719	1718	166	47, 79, 91	RI, MS	-	-	0.2	-	-	-	-	-
Monoterpene hydrocarbons						83.6	85.6	83.9	78.2	85.8	91.6	69.4	43.8
Oxygenated monoterpenes						6.3	3.0	4.6	6.3	4.5	0.2	9.7	3.7
Sesquiterpene hydrocarbons						3.5	6.9	4.2	1.8	8.3	5.5	10.2	36.0
Oxygenated sesquiterpenes						1.8	1.5	1.8	2.3	0.6	-	4.9	3.0
Total identified						95.2	97.0	94.5	88.6	99.2	97.3	94.2	86.5

^a^ Elution order on DB-5 column according to C_9_–C_24_ *n*-alkanes; ^b^ RI (Lit.)—Literature retention indices; ^c^ RI (Cal.)—Retention indices relative to *n*-alkanes on HP-5MS column; M^+^ (m/z) = molecular ion peak; Fragment ions (m/z) = characteristic ions obtained from electron impact mass fragmentation; IM = Identification method based on MS library matching and retention index (RI) comparison;—Not identified; t-trace.

**Table 3:** Antibacterial activity of *Senecio* species essential oils (Zones of inhibition) *^,^,<^.

Code	Bc ^^^,$^	Bp	Sa1	Sa2	Ef	Ec	*E. coli*	Kp	Pv1	Pv2	Pa	Sm
S1	13.0 ± 1.0 ^b^	12.7 ± 1.5 ^b^	21.7 ± 0.6 ^c^	21.0 ± 1.0 ^d^	15.0 ± 1.0 ^d^	7.3 ± 1.2 ^c^	15.7 ± 0.6 ^c^	12.0 ± 1.0 ^b^	8.3 ± 0.6 ^c^	9.7 ± 0.6 ^c^	10.7 ± 0.6 ^c^	9.0 ± 0.0 ^c^
S2	12.7 ± 1.6 ^b^	14.0 ± 1.0 ^bc^	15.7 ± 0.6 ^d^	13.3 ± 0.6 ^e^	11.7 ± 0.6 ^e^	6.0 ± 0.0 ^c^	12.7 ± 0.6 ^d^	8.7 ± 1.2 ^c^	6.3 ± 0.6 ^d^	7.3 ± 0.6 ^d^	7.7 ± 1.2 ^d^	8.3 ± 0.6 ^c^
S3	12.6 ± 1.2 ^b^	13.3 ± 0.6 ^bc^	19.3 ± 0.6 ^c^	18.7 ± 0.6 ^c^	16.0 ± 1.0 ^c^	7.0 ± 0.0 ^c^	15.3 ± 0.6 ^c^	11.7 ± 0.6 ^b^	8.0 ± 0.0 ^c^	8.3 ± 0.6 ^c^	9.0 ± 0.0 ^c^	8.7 ± 0.6 ^c^
S4	9.7 ± 0.6 ^c^	9.0 ± 1.0 ^c^	13.3 ± 0.6 ^e^	12.3 ± 0.6 ^f^	12.0 ± 1.0 ^e^	6.6 ± 0.6 ^c^	11.3 ± 0.6 ^d^	11.0 ± 1.0 ^b^	6.7 ± 0.6 ^d^	7.0 ± 1.0 ^d^	7.0 ± 1.0 ^d^	7.3 ± 0.6 ^c^
S5	8.7 ± 0.6 ^c^	8.3 ± 0.6 ^c^	11.3 ± 0.6 ^f^	10.7 ± 0.6 ^f^	8.7 ± 1.2 ^f^	6.0 ± 0.0 ^c^	9.3 ± 0.6 ^e^	8.0 ± 1.0 ^c^	6.6 ± 0.6 ^d^	7.0 ± 1.0 ^d^	7.3 ± 0.6 ^d^	7.0 ± 1.0 ^c^
S6	10.7 ± 0.6 ^c^	10.3 ± 0.6 ^c^	10.7 ± 0.6 ^f^	9.3 ± 0.6 ^f^	8.3 ± 0.6 ^f^	6.0 ± 0.0 ^c^	10.0 ± 0.0 ^e^	6.7 ± 0.6 ^c^	6.0 ± 0.0 ^d^	6.3 ± 0.6 ^d^	6.0 ± 0.0 ^d^	6.3 ± 0.6 ^c^
S7	11.0 ± 1.0 ^c^	10.7 ± 0.6 ^c^	12.3 ± 0.6 ^e^	12.7 ± 0.6 ^e^	9.7 ± 1.5 ^f^	6.3 ± 0.6 ^c^	10.3 ± 0.6 ^e^	6.3 ± 0.6 ^c^	6.0 ± 0.0 ^d^	6.7 ± 0.6 ^d^	6.3 ± 0.6 ^d^	7.0 ± 1.0 ^c^
S8	21.0 ± 1.0 ^a^	15.0 ± 1.0 ^a^	23.7 ± 0.6 ^a^	20.1 ± 1.2 ^d^	16.3 ± 0.6 ^c^	8.3 ± 0.6 ^c^	15.3 ± 1.5 ^c^	12.0 ± 1.0 ^b^	7.3 ± 0.6 ^c^	8.7 ± 0.6 ^c^	10.0 ± 1.0 ^c^	9.0 ± 1.0 ^c^
Chl ^%^	23.7 ± 1.3 ^a^	16.3 ± 1.3 ^a^	16.7 ± 1.3 ^d^	13.7 ± 1.3 ^e^	20.3 ± 1.3 ^b^	13.3 ± 1.3 ^a^	23.7 ± 1.3 ^a^	20.0 ± 1.4 ^a^	6.0 ± 0.0 ^d^	21.0 ± 2.0 ^a^	22.7 ± 1.7 ^a^	6.0 ± 0.0 ^c^
Gent ^£^	14.0 ± 2.0 ^b^	13.3 ± 1.7 ^bc^	17.3 ± 0.9 ^c^	14.3 ± 1.3 ^e^	16.0 ± 1.6 ^c^	17.7 ± 0.5 ^b^	21.3 ± 1.3 ^b^	23.7 ± 1.3 ^a^	6.0 ± 0.0 ^d^	21.3 ± 0.9 ^a^	20.7 ± 0.9 ^a^	14.3 ± 0.5 ^b^
Tetra ^&^	13.3 ± 1.3 ^b^	14.0 ± 1.3 ^bc^	18.7 ± 0.9 ^c^	ND	ND	13.0 ± 1.4 ^b^	23.0 ± 1.4 ^a^	17.6 ± 1.3 ^a^	6.0 ± 0.0 ^d^	6.0 ± 0.0 ^d^	14.7 ± 0.5 ^c^	15.7 ± 1.3 ^b^

* Inhibition zone diameters (mm) -including diameter of sterile disc (6 mm); ^^^ Values given as mean ± SD (3 replicates). ^<^ *Essential oil* tested at 10 µL/disc. ^^^^ Bacteria: *Bc*-*B. cereus* (ATCC 10702), *Bp*-*B. pumilus* (ATCC 14884), *Sa1*-*S. aureus* (ATCC 3983), *Sa2*-*S. aureus* (ATCC 6538), *Ef*-*E. faecalis* (ATCC 29212), *Ec*-*E. cloacae* (ATCC 13047), *E.coli*-*E. coli* (ATCC 4983), *Kp*-*K. pneumoniae* (ATCC 2983), *Pv1*-*P. vulgaris* (ATCC 6830), *Pv2*-*P. vulgaris* (CSIR 0030), *Pa*-*P. aeruginosa* (ATCC19582) and *Sm*-*S. marcescens* (ATCC 9986). ND = Not Determined; ^$^ Means within each column followed by different superscript letters (a–f) are assigned based, often from highest activity (a) to lowest (f), depending on the dataset and values sharing the same letter are not significantly different from each other *p* > 0.05, and different letters are significantly different *p* < 0.05, (one-way ANOVA followed by Tukey’s HSD test). ^%^ Chl-Chloramphenicol; ^£^ Gent-Gentamycin; ^&^ Tetra-Tetreacycline. ATCC = American Type Culture Collection, USA. CSIR = Council for Scientific and Industrial Research, South Africa.

**Table 4:** Minimum inhibitory concentrations of *Senecio* species essential oils ^^^.

Code	Bc *^,$^	Bp	Sa1	Sa2	Sf	Ec	*E.coli*	Kp	Pv1	Pv2	Pa	Sm
S1	2.5 ^b^	2.5 ^b^	0.08 ^a^	0.08 ^a^	2.5 ^c^	10 ^a^	2.5 ^b^	2.5 ^c^	10 ^a^	5 ^b^	2.5 ^b^	2.5 ^b^
S2	1.25 ^c^	1.25 ^c^	1.25 ^c^	2.5 ^b^	2.5 ^c^	10 ^a^	1.25 ^c^	5 ^b^	10 ^a^	10 ^a^	10 ^a^	5 ^a^
S3	1.25 ^c^	1.25 ^c^	0.31 ^b^	0.31 ^b^	1.25 ^b^	10 ^a^	0.08 ^a^	1.25 ^b^	10 ^a^	5 ^b^	1.25 ^c^	1.25 ^c^
S4	5 ^a^	5 ^a^	1.25 ^c^	1.25 ^c^	2.5 ^c^	10 ^a^	1.25 ^c^	5 ^b^	10 ^a^	5 ^b^	2.5 ^b^	1.25 ^c^
S5	2.5 ^b^	5 ^a^	2.5 ^b^	2.5 ^b^	5 ^a^	10 ^a^	2.5 ^b^	5 ^b^	10 ^a^	10 ^a^	5 ^a^	5 ^a^
S6	2.5 ^b^	2.5 ^b^	5 ^a^	5 ^a^	5 ^a^	10 ^a^	5 ^a^	10 ^a^	10 ^a^	10 ^a^	10 ^a^	10 ^a^
S7	1.25 ^c^	5 ^a^	1.25 ^c^	1.25 ^c^	2.5 ^c^	10 ^a^	2.5 ^b^	10 ^a^	10 ^a^	5 ^b^	5 ^a^	5 ^a^
S8	0.31 ^a^	0.63 ^c^	0.08 ^a^	0.08 ^a^	1.25 ^b^	10 ^a^	1.25 ^c^	2.5 ^c^	10 ^a^	5 ^b^	2.5 ^b^	2.5 ^b^
Chl	0.08 ^a^	0.63 ^c^	0.31 ^b^	0.31 ^b^	0.16 ^a^	5 ^b^	0.08 ^a^	0.63 ^a^	ND	0.63 ^a^	0.31 ^a^	ND
Gent	0.63 ^b^	1.25 ^c^	0.31 ^b^	0.63 ^b^	1.25 ^b^	2.5 ^c^	0.16 ^b^	0.08 ^a^	ND	0.63 ^a^	0.63 ^a^	1.25 ^a^
Tetra	1.25 ^c^	1.25 ^c^	0.31 ^b^	0.31 ^b^	ND	2.5 ^c^	0.31 ^b^	0.63 ^a^	ND	–	0.63 ^a^	0.63 ^a^

^^^ MIC values are given in mg/mL; * Bacteria as stated in Table 3; ^$^ Means within each column followed by different superscript letters (a–c) are significantly different at *p* < 0.05 are not significantly different from each other *p* > 0.05, (one-way ANOVA followed by Tukey’s HSD test). ND: not determined.

**Table 5:** Antioxidant activity of *S. polyanthemoides* essential oils ^a^.

Sample	Antioxidant ^^^		
	DPPH ^b^	OH ^c^	NO ^d^
S1	>250	<10	16.46
S2	>250	32.87	155.80
S3	>250	11.53	101.67
S4	>250	50.87	49.27
S5	>250	<10	153.40
S6	>250	<10	101.67
S7	219.32	<10	32.07
S8	>250	<10	41.13
BHA ^e^	10.70	<10	<10
BHT ^f^	<10	<10	<10
AA ^g^	<10	<10	15.19
TCP ^h^	<10	<10	11.96

^a^ Mean ± SE (*n* = 3); ^^^ Values represent IC_50_ (µg/mL) and lower values indicate stronger antioxidant activity; ^b^ DPPH = 2,2-diphenyl-1-picrylhydrazyl radical scavenging assay; ^c^ OH = hydroxyl radical scavenging assay; ^d^ NO = nitric oxide scavenging assay; ^e^ BHA—Butyl hydroxyl anisole; ^f^ BHT—butyl hydroxyl toluene; ^g^ AA—Ascorbic acid; ^h^ TCP—α-Tocopherol.

**Table 6:** Reducing power of essential oils of *Senecio* species and standard antioxidants *^,^^.

Sample	10	20	50	100	250
S1	0.063 ± 0.002 ^b^	0.067 ± 0.001 ^b^	0.071 ± 0.001 ^b^	0.080 ± 0.002 ^c^	0.100 ± 0.001 ^d^
S2	0.082 ± 0.002 ^d^	0.088 ± 0.002 ^d^	0.099 ± 0.001 ^d^	0.110 ± 0.002 ^d^	0.125 ± 0.001 ^e^
S3	0.076 ± 0.002 ^c^	0.080 ± 0.001 ^c^	0.086 ± 0.002 ^c^	0.101 ± 0.001 ^d^	0.115 ± 0.002 ^d^
S4	0.070 ± 0.001 ^c^	0.076 ± 0.001 ^c^	0.086 ± 0.002 ^c^	0.097 ± 0.002 ^c^	0.116 ± 0.001 ^d^
S5	0.070 ± 0.004 ^c^	0.086 ± 0.003 ^d^	0.092 ± 0.006 ^d^	0.126 ± 0.002 ^e^	0.145 ± 0.002 ^e^
S6	0.063 ± 0.003 ^b^	0.072 ± 0.001 ^b^	0.077 ± 0.001 ^b^	0.084 ± 0.001 ^c^	0.125 ± 0.002 ^e^
S7	0.080 ± 0.001 ^d^	0.097 ± 0.002 ^e^	0.101 ± 0.002 ^e^	0.110 ± 0.002 ^d^	0.140 ± 0.004 ^e^
S8	0.055 ± 0.004 ^a^	0.060 ± 0.002 ^a^	0.064 ± 0.004 ^a^	0.071 ± 0.002 ^a^	0.083 ± 0.002 ^a^
BHA ^^^^	0.058 ± 0.002 ^ab^	0.072 ± 0.004 ^b^	0.081 ± 0.003 ^b^	0.096 ± 0.003 ^c^	0.174 ± 0.003 ^f^
BHT	0.057 ± 0.000 ^a^	0.069 ± 0.001 ^b^	0.075 ± 0.001 ^b^	0.081 ± 0.001 ^c^	0.154 ± 0.002 ^e^
AA	0.159 ± 0.002 ^f^	0.171 ± 0.003 ^f^	0.188 ± 0.002 ^f^	0.194 ± 0.003 ^f^	0.242 ± 0.003 ^g^
TCP	0.058 ± 0.003 ^ab^	0.066 ± 0.003 ^b^	0.075 ± 0.002 ^b^	0.091 ± 0.002 ^c^	0.166 ± 0.002 ^f^

* Test concentrations ranged from 10 to 250 µg/mL; ^^^ Values are expressed as mean ± standard deviation (*n* = 3); Means within each column followed by different superscript letters (a–g) are assigned based, often from highest activity (a) to lowest (g), depending on the dataset. Values sharing the same letter are not significantly different from each other *p* > 0.05, and different letters are significantly different *p* < 0.05, (one-way ANOVA followed by Tukey’s HSD test). ^^^^ (BHA, BHT, AA and α-Tocopherol)-as stated in Table 5.

**Table 7:** Brine shrimp lethality assay of *S. polyanthemoides* essential oils *.

Sample	LC_50_ (µg/mL) (95% CI)	LC_90_ (µg/mL) (95% CI)
S1	13.13 ^b^ (6.51–19.53)	68.33 ^c^ (50.82–91.87)
S2	13.83 ^b^ (7.05–20.50)	72.74 ^c^ (53.97–98.04)
S3	16.52 ^a^ (8.81–24.54)	94.85 ^a^ (69.15–130.12)
S4	11.45 ^c^ (5.29–17.12)	70.68 ^c^ (51.47–97.07)
S5	11.37 ^c^ (5.54–16.56)	47.32 ^b^ (36.41–61.51)
S6	9.63 ^c^ (3.53–15.29)	54.57 ^b^ (40.65–73.24)
S7	11.75 ^c^ (6.59–16.17)	38.35 ^b^ (30.41–48.37)
S8	14.64 ^a^ (8.35–20.84)	63.51 ^c^ (48.52–83.13)
1% DMSO	59.70 (29.62–130.86)	—
Artificial seawater	>250	>250
Distilled water	>250	>250

* Values are expressed as LC_50_ and LC_90_ with 95% confidence intervals. Different superscript letters (a–c) within a column indicate significant differences (*p* < 0.05). Artificial seawater and distilled water showed no mortality; therefore, LC_50_ and LC_90_ values were not estimable and are reported as >250 µg/mL. The solvent control (1% DMSO) exhibited low toxicity; LC_90_ could not be reliably estimated.

**Table 8:** Larvicidal activity of *S. polyanthemoides* essential oils against *Culex quinquefasciatus*.

Sample	LC_50_ ^b^	LC_90_ ^b^	Slope ± SE	χ^2^ (df)	Potency Group ^
S1	50.70	170.69	1.88 ± 0.33	1.89	(3) ^d^
S2	39.68	131.17	2.12 ± 0.27	1.38	(3) ^bc^
S3	42.77	145.05	2.08 ± 0.29	1.51	(3) ^bc^
S4	47.20	159.44	1.95 ± 0.31	1.72	(3) ^cd^
S5	30.70	131.73	2.18 ± 0.28	1.45	(3) ^ab^
S6	42.77	145.05	2.08 ± 0.29	1.51	(3) ^bc^
S7	36.31	118.84	2.41 ± 0.32	1.12	(3) ^a^
S8	33.70	161.08	2.05 ± 0.30	1.63	(3) ^ab^
1% DMSO ^c^	NA	NA	NA	NA	No effect
Distilled water ^c^	NA	NA	NA	NA	NO effect

^a^ values (µg/mL), ^b^ LC_50_ and LC_90_ were estimated using probit analysis. Regression parameters include slope ± standard error and chi-square (χ^2^) goodness-of-fit values (df = 3). Lower LC values indicate higher larvicidal potency. ^c^ Control treatments, including 1% Dimethyl sulfoxide (DMSO) and distilled water, showed no larvicidal activity, confirming that mortality was due to the essential oils. ^ Potency group—different superscript letters indicate statistically significant differences among samples (*p* < 0.05).

## Data Availability

The original contributions presented in this study are included in the article/Supplementary Material. Further inquiries can be directed to the corresponding author.

## Supplementary Materials

The following supporting information can be downloaded at: https://www.mdpi.com/article/10.3390/molecules31122006/s1, Figures S1–S8: GC/MS Chromatograms of *S. polyanthemoides* essential oils.
