# Supplementary material for: Senecio polyanthemoides Sch. Bip. (Asteraceae) Essential Oils: Chemical Composition, Interpopulation Variability Study and In Vitro Biological Activities of Eight Wild Populations
Source: Molecules. 2026 Jun 8;31(12):2006. doi: 10.3390/molecules31122006 (PMC13305831; doi:10.3390/molecules31122006)
Supplement: Supplementary file 1 [file molecules-31-02006-s001.zip › molecules-4267020-supplementary.pdf]

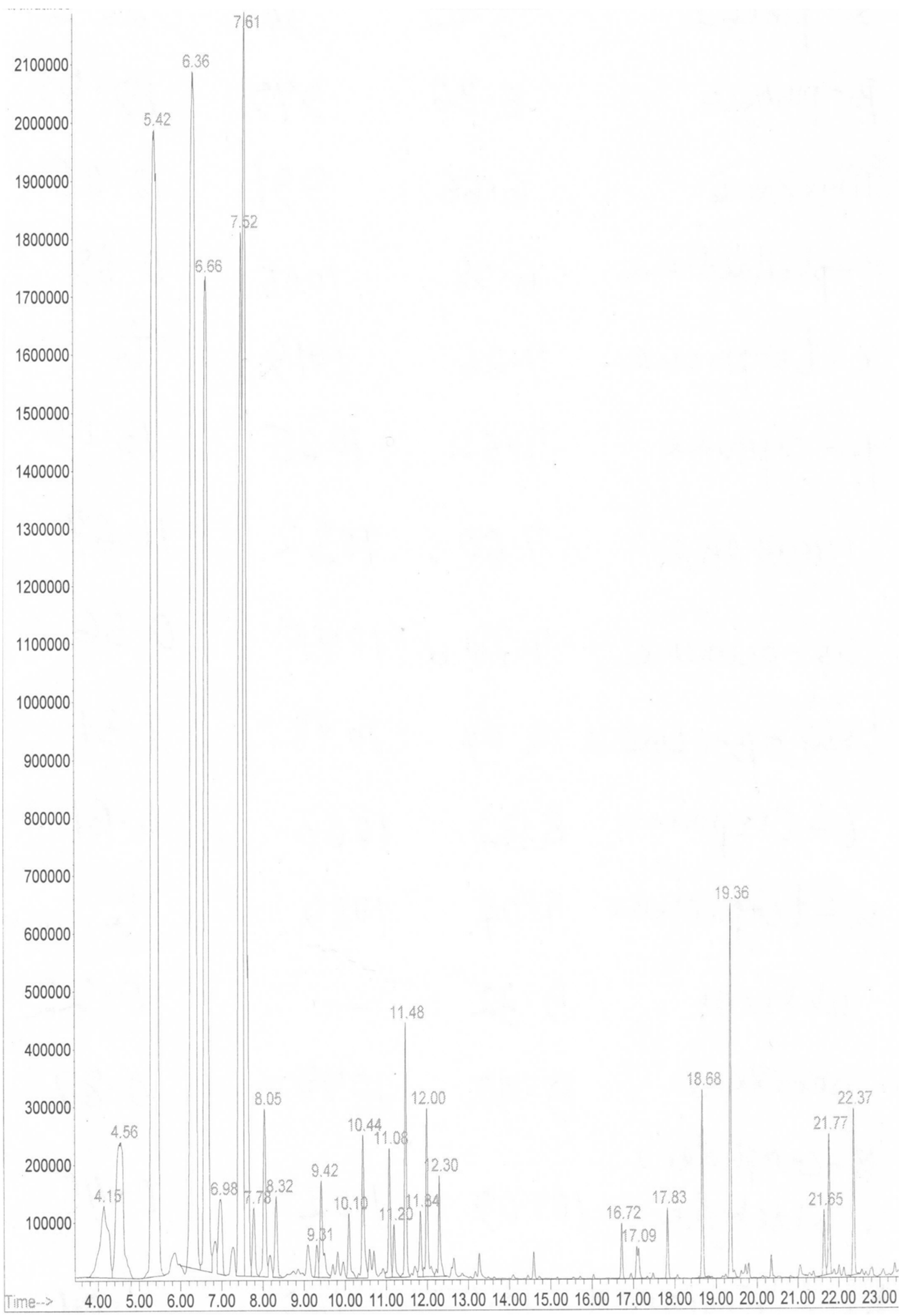


Figure S1 GC/MS Chromatogram of *Senecio polyanthemoides* essential oil (S1)

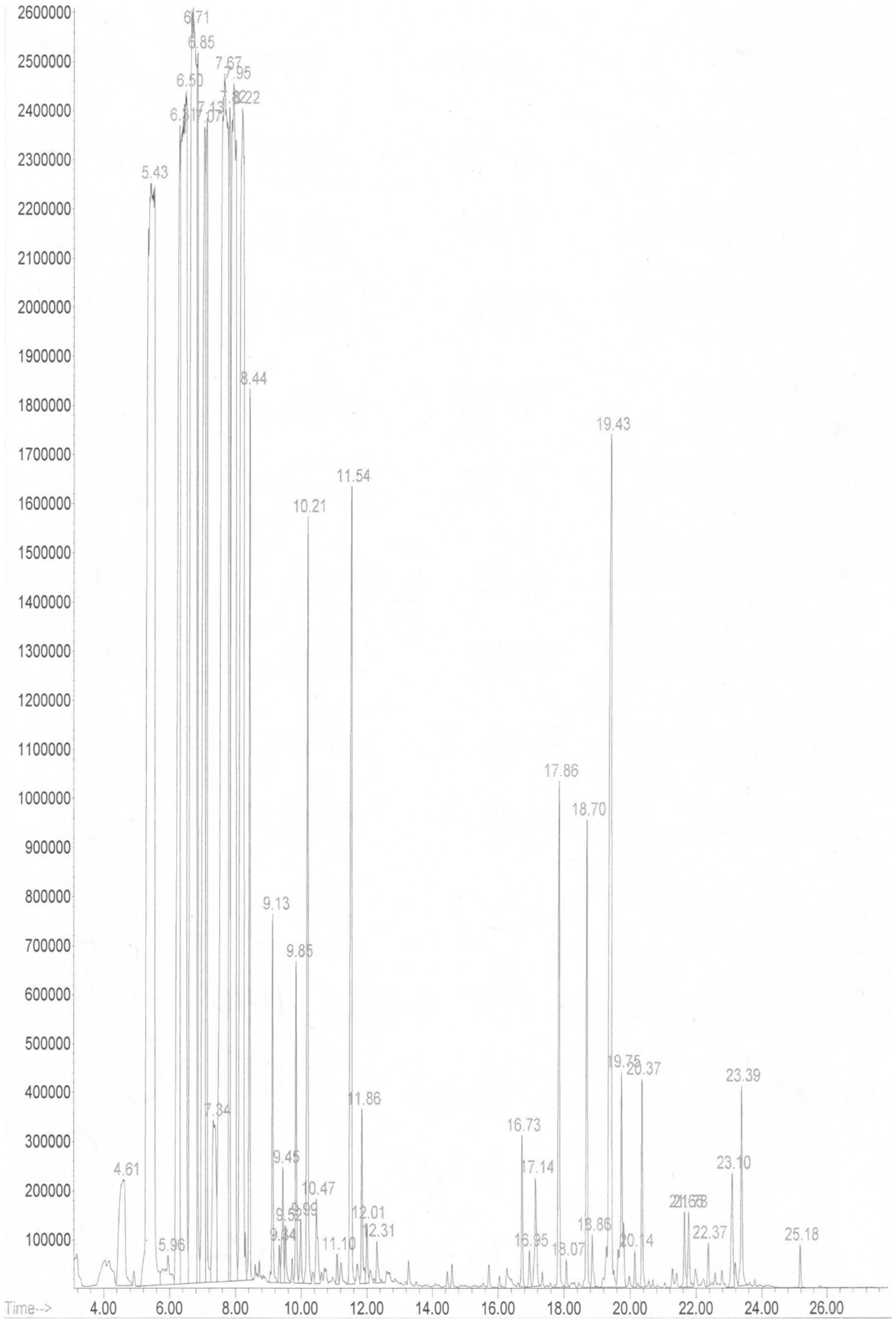


Figure S2 GC/MS Chromatogram of *Senecio polyanthemoides* essential oil (S2)

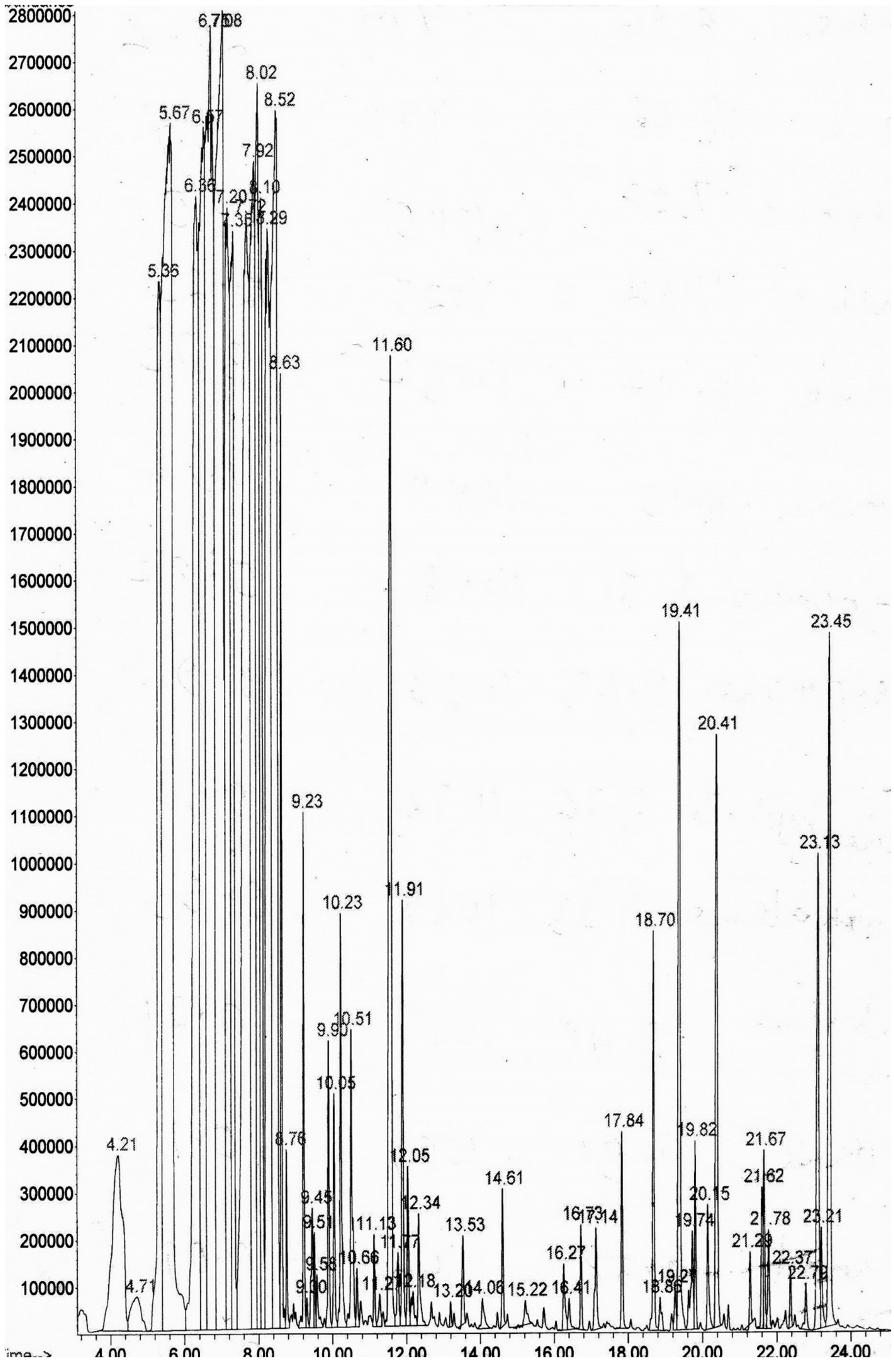

Figure S3 GC/MS Chromatogram of *S. polyanthemoides* (S3)

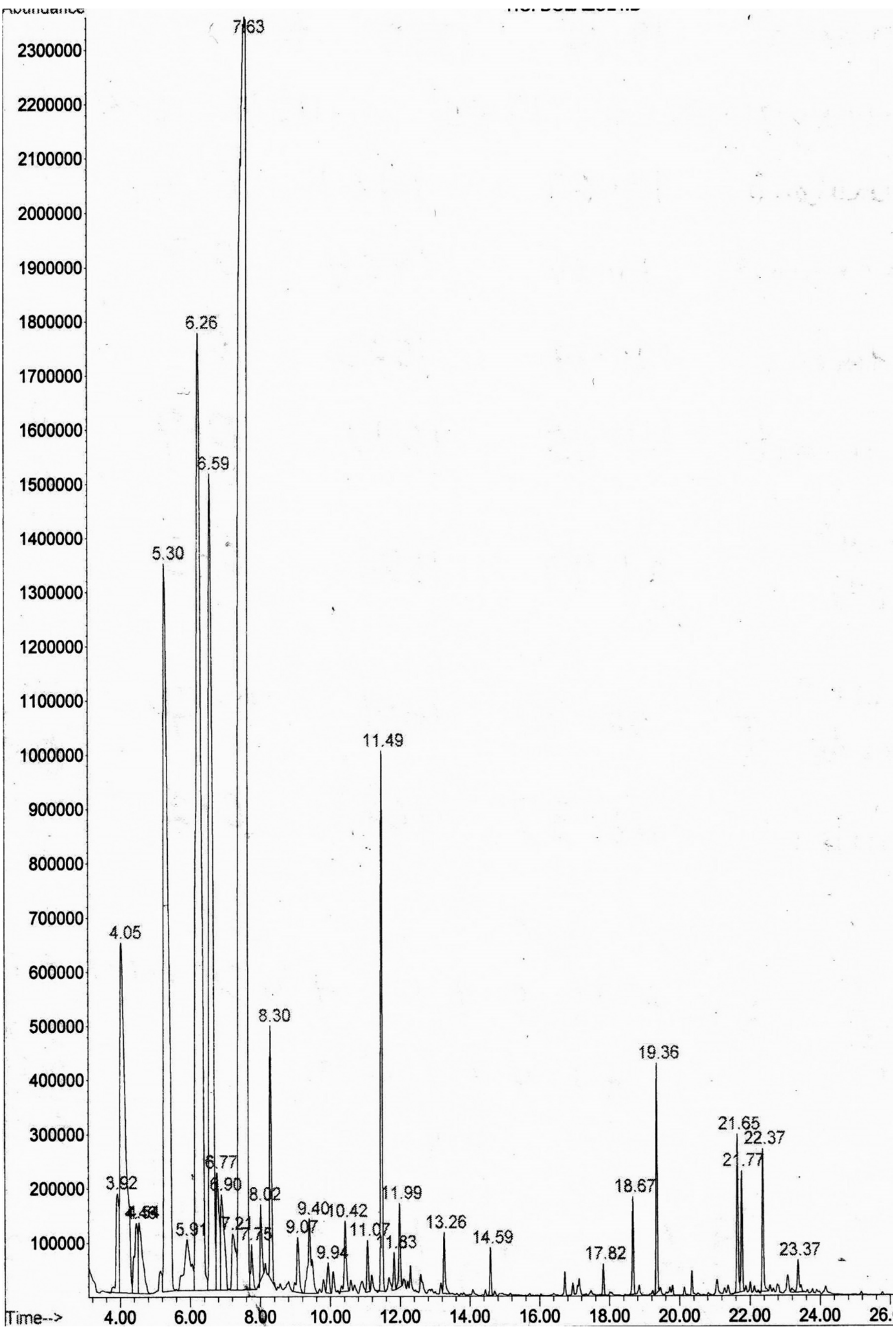


Figure S4 GC/MS Chromatogram of *S. polyanthemoides* (S4)

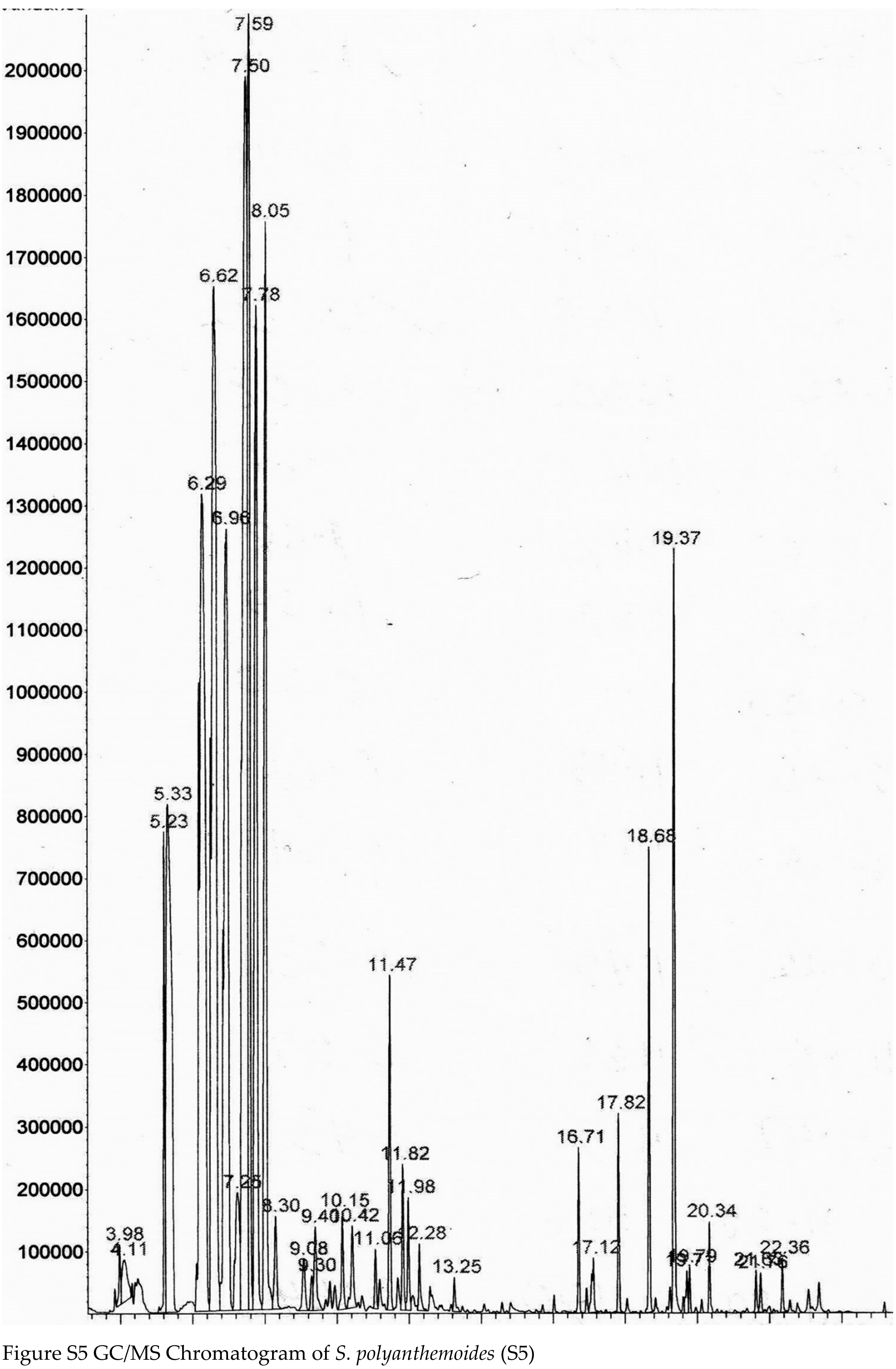


Figure S5 GC/MS Chromatogram of *S. polyanthemoides* (S5)

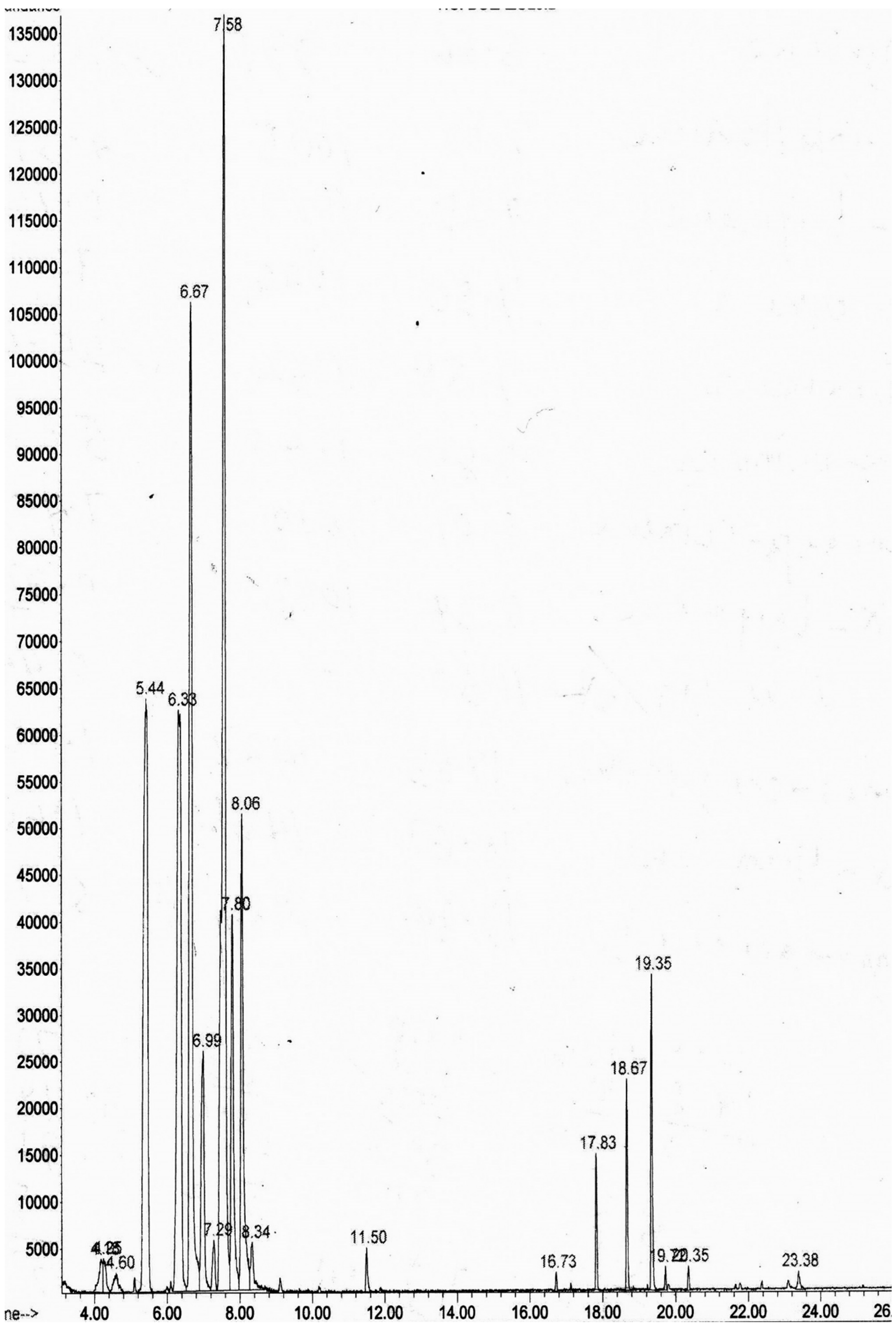


Figure S6 GC/MS Chromatogram of *S. polyanthemoides* (S6)

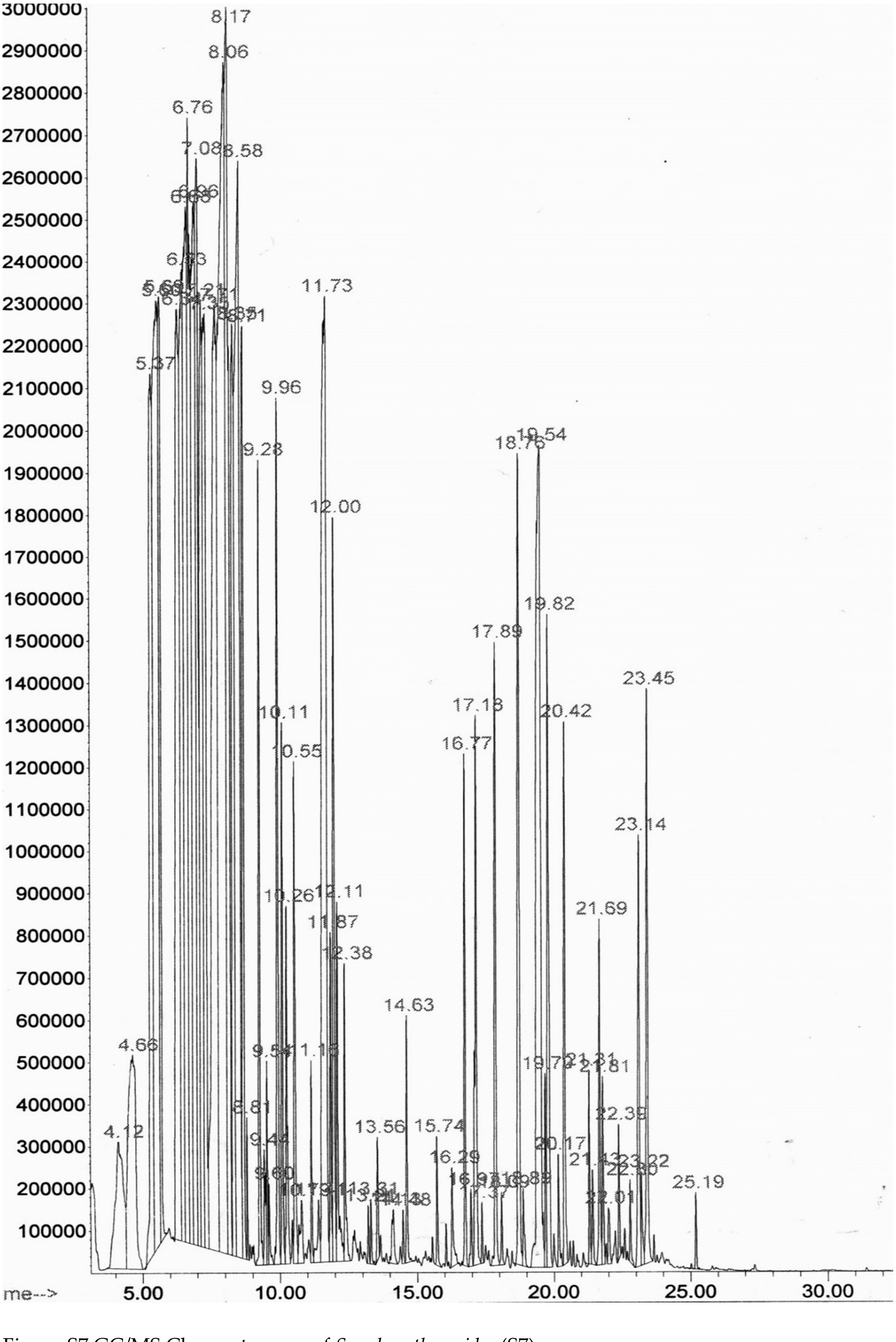


Figure S7 GC/MS Chromatogram of *S. polyanthemoides* (S7)

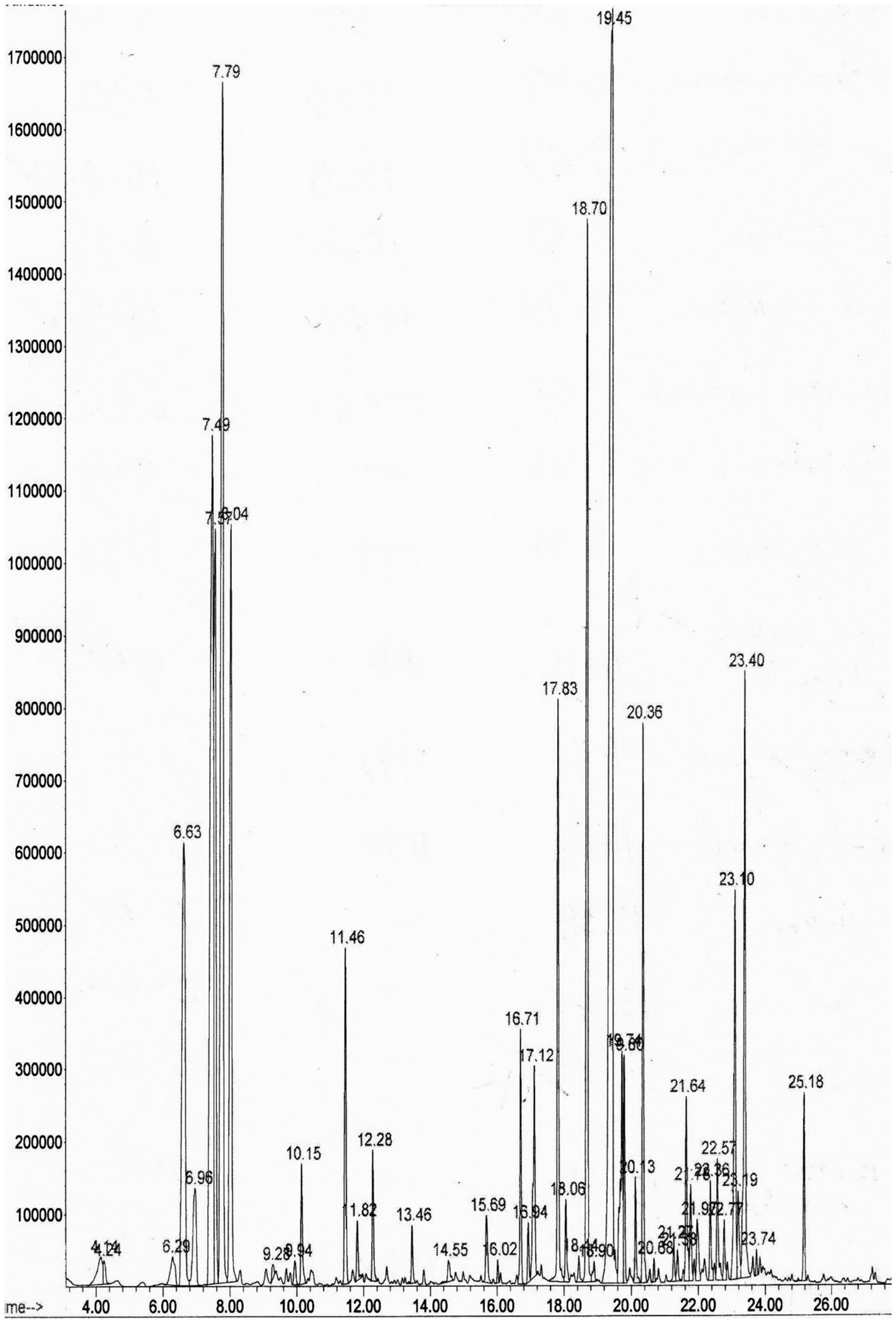


Figure S8 GC/MS Chromatogram of *S. polyanthemoides* (S8)
